# Phenolic Endocrine-Disrupting Chemical Exposure and Systemic Biomarker Variability in Patients with Lung Cancer

**DOI:** 10.3390/medicina62071409

**Published:** 2026-07-21

**Authors:** Larisa Đurić, Nataša Milošević, Maja Milanović, Danica Sazdanić-Velikić, Jana Pavlović, Milorad Španović, Nataša Milić

**Affiliations:** 1 Faculty of Medicine, Department of Pharmacy, University of Novi Sad, 21000 Novi Sad, Serbia; larisa.djuric@mf.uns.ac.rs (L.Đ.); natasa.milosevic@mf.uns.ac.rs (N.M.); natasa.milic@mf.uns.ac.rs (N.M.); 2 Faculty of Medicine, Institute for Pulmonary Diseases of Vojvodina, Clinic for Pulmonary Oncology, University of Novi Sad, 21204 Sremska Kamenica, Serbia; danica.sazdanic-velikic@mf.uns.ac.rs; 3 Faculty of Medicine Foča, University of East Sarajevo, 73300 Foča, Bosnia and Herzegovina; jana.stojanovic@ues.rs.ba; 4 Faculty of Medicine, Institute of Public Health of Vojvodina, University of Novi Sad, 21000 Novi Sad, Serbia; milorad.spanovic@mf.uns.ac.rs

**Keywords:** exposome, environmental oncology, endocrine-disrupting chemicals, cardiometabolic parameters, triclosan

## Abstract

*Background and Objectives*: Environmental exposure to endocrine-disrupting chemicals (EDCs) is increasingly recognized as a potential contributor to cancer-related biological variability; however, human biomonitoring data in oncology populations remain limited. The present study aimed to assess urinary concentrations of selected phenolic EDCs and their associations with cardiometabolic, hematological, inflammatory, and survival-related parameters in patients with advanced lung cancer. *Materials and Methods*: A total of 190 patients diagnosed with stage IIIB/IV lung cancer were included in this study. Urinary concentrations of bisphenol A (BPA), bisphenol S (BPS), triclosan (TCS), and resorcinol (RCO) were determined using validated analytical methods. Associations between exposure biomarkers and clinical laboratory parameters were evaluated using sex-stratified statistical analyses and regression models adjusted for age and body mass index. *Results*: TCS was the most frequently quantified compound (29.47%), followed by BPS (27.37%), RCO (11.58%), and BPA (7.89%). Higher odds of TCS quantification were observed in patients with lung adenocarcinoma and a higher probability of BPA quantification in patients with squamous-cell carcinoma. Sex-specific exposure patterns were observed, with higher BPA and BPS concentrations measured among female patients. Exposure to phenolic EDCs was associated with alterations in kidney function biomarkers, liver enzyme activity, inflammatory cell profiles, and anthropometric indicators. In particular, BPA and BPS showed associations with renal function markers and systemic inflammatory parameters, while TCS exposure was related to reduced leukocyte subpopulations. Survival analysis demonstrates borderline associations for BPA between exposure groups. *Conclusions*: These findings provide novel human biomonitoring evidence linking exposure to phenolic endocrine-disrupting chemicals with systemic metabolic and inflammatory variability in patients with advanced lung cancer. The observed associations support the biological plausibility that environmental endocrine disruptors may contribute to interindividual heterogeneity in cancer-related physiological responses.

## 1. Introduction

The carcinogenic potential of environmental pollutants varies depending on pollution sources, climatic conditions, and regional topography. Exposure to the pollutants substantially increases cancer risk, and lung cancer is associated not only with smoking but also with air pollution, radon exposure, second-hand smoke, industrial chemicals, and other environmental contaminants [1]. There is a growing global public health concern regarding the increasing contribution of environmental and non-tobacco-related risk factors to lung cancer incidence. Lung cancer in never-smokers accounts for approximately 25% of all lung cancer cases worldwide [2]. Nearly half of the lung cancer patients are diagnosed at an advanced stage (IIIB/IV), when curative options are limited [3].

Considering the distinct histological subtypes of lung cancer with different etiological profiles, adenocarcinoma is more often recorded in non-smokers or people with a mild history of smoking [4,5], making environmental factors of particular interest. In contrast, squamous-cell carcinoma and small-cell lung cancer are strongly associated with tobacco smoking and have a different molecular profile [6,7]. Recognition of these differences in histological subtypes may indicate the variable sensitivity of different tumor types to environmental factors, including endocrine-disrupting chemicals (EDCs). The mechanisms of EDCs role in lung cancer have not yet been elucidated.

EDCs can mimic, block, or interfere with hormonal signaling pathways in the human endocrine system, and there is growing evidence about their adverse health effects including metabolic disorders, reproductive dysfunction, and certain cancers [8,9,10,11,12]. Their mechanism of adverse effects also involves induced oxidative stress, chronic inflammation, and epigenetic alterations [8,9,10,11,12]. Bisphenols are wide-spread chemicals used in the production of polycarbonate plastics and epoxy resins. The most abundant compound, bisphenol A (BPA), can be found in plastic bottles, food can coatings, dental materials, medical devices, toys, thermal paper, etc. Its analogue, bisphenol S (BPS), is commonly used in thermal paper, can coating, epoxy adhesives, and dye additives [13]. BPA exposure has been associated with breast, ovarian, thyroid, and colon cancer, as well as with nasopharyngeal carcinomas, hematologic malignancies and osteosarcoma [14]. Similarly, BPS exposure has been linked to breast [15], ovarian [16], and prostate cancers [17]. There are some findings indicating the influence of BPA on lung cancer progression by promoting migration and invasion of lung cancer cells in vitro [18], while other reports suggested its possible role in lung adenocarcinoma [19]. Moreover, elevated BPA levels have also been reported in patients with non-small-cell lung cancer (NSCLC) compared with controls, suggesting a possible association between environmental exposure and lung cancer risk [20]. Evidence regarding the role of BPS in lung carcinogenesis remains limited; however, experimental studies suggest its potential involvement in NSCLC development [21]. Triclosan (TCS) is a synthetic, antimicrobial agent, recognized as an EDC [22] and widely used in various products including disinfectants, soaps, mouthwashes, toothpastes, deodorants, as well as in clothing textiles and furniture materials [23]. TCS exposure has been associated with breast [24], esophageal [25], and skin cancers [26]. Recent studies also suggested a possible role in lung cancer progression, as TCS promotes epithelial-to-mesenchymal transition in aggressive, anoikis-resistant human H460 lung cancer cells. In addition, TCS significantly increases colony formation in tumorigenicity assays and induces mesenchymal-like morphological changes accompanied by reduced cell–cell adhesion [27]. An animal model further demonstrated that TCS exposure may induce marked structural abnormalities in the lungs, including atypical alveolar cells, interstitial hyperplasia, and thickened vascular walls. Moreover, extensive pulmonary fibrosis, characterized by increased collagen deposition, fibroblast proliferation, and myofibroblast accumulation, has been observed in the offspring of TCS-exposed mice [28]. In addition, TCS interferes with multiple endocrine-signaling pathways, including steroidogenesis and thyroid hormone regulation, supporting its classification as a biologically active endocrine disruptor with potential relevance for carcinogenesis [23]. Resorcinol (RCO) is a ubiquitous chemical present in personal care products including hair dyes, anti-acne preparations, and peels, as well as in rubber products, wood adhesives, flame retardants, UV stabilizers, and dyes [29]. It is assumed that RCO disrupts thyroid gland function, although there are limited data about its effects on cancer development and progression [30,31]. Derivatives of RCO are even observed as antitumor agents, while the effect of RCO on lung cancer remains largely unexplored [32,33].

Biomonitoring is a modern and highly relevant approach for assessing human exposure to EDCs, as it enables trace detection directly in biological samples [34]. In the context of lung cancer, the application of this approach in a cohort of pathohistologically confirmed patients enables a unique assessment of the potential association between interindividual exposure to selected EDCs and disease parameters, taking into account gender differences and histological tumor subtypes. To the best of our knowledge, this is the first study to simultaneously assess urinary levels of four environmental pollutants: TCS, RCO, BPA and BPS in patients with different types of advanced stage (IIIB/IV) lung cancer. This study aimed to investigate the associations between urinary levels of selected EDCs and cardiometabolic, liver, kidney and inflammatory parameters, hematological status and immune response indicators, as well as anthropometric characteristics.

## 2. Material and Methods

### 2.1. Study Population

A total of over 400 patients referred to the Institute for Pulmonary Disease of Vojvodina, Sremska Kamenica, Serbia, with suspected lung cancer were screened for eligibility. Following diagnostic evaluation and histopathological confirmation, 190 patients (105 men and 85 women) with advanced stage IIIB or IV lung cancer were included in this study. Inclusion criteria included patients age over 18 years, creatinine clearance >60 mL/min as calculated using the Cockcroft–Gault formula, histologically confirmed advanced-stage (IIIB or IV) lung cancer, and written informed consent. All participants had an Eastern Cooperative Oncology Group (ECOG) performance status of 0 or 1. Patients who were pregnant or had a previous history of malignancy or were diagnosed with lung cancer stage below IIIB were not included in this study. Patient enrollment was conducted between March 2022 and April 2023. Morning urine samples were collected in polystyrene plastic containers within 24 h of hospitalization and prior to initiation of oncological treatment. Urinary creatinine concentrations were determined using the Jaffé method on a Beckman Coulter AU480 biochemical analyzer (Beckman Coulter, Inc., Brea, CA, USA). The results were expressed in µmol/L, and converted to g/L as needed. All subjects underwent standard anthropometric measurements, including body mass (kg), height (m), waist and hip circumference (cm). Body mass was measured using a medical digital scale, while body height was measured with a Martin anthropometer. Based on the obtained values, body mass index (BMI) was calculated as a quotient of body mass and the square of height. Waist-to-height ratio (WtHR) and waist-to-hip ratio (WHR) were also calculated. Blood pressure (BP), expressed in millimeters of mercury (mmHg), and pulse rate, expressed in beats per minute (bpm), were measured with a standard sphygmomanometer (65 mm). Venous blood samples were taken after a 12 h fast. Biochemical parameters, including alanine aminotransferase (ALT), aspartate aminotransferase (AST), gamma-glutamyl transferase (GGT), lactate dehydrogenase (LDH), alkaline phosphatase (ALP), total and direct bilirubin, urea, creatinine, creatine kinase (CK), creatine kinase MB fraction (CK-MB), uric acid, glucose, and C-reactive protein (CRP), were determined spectrophotometrically from serum using a Cobas automated biochemical analyzer c311 (Roche Diagnostics, Mannheim, Germany). Complete blood count with leukocyte count was obtained using a Beckman Coulter DxH 900 hematology analyzer (Beckman Coulter, Inc., Miami, FL, USA). The analyzed parameters included erythrocyte parameters: red blood cell count (RBC), hemoglobin concentration (Hgb), hematocrit (Hct), mean corpuscular volume (MCV), mean corpuscular hemoglobin (MCH) and average hemoglobin concentration per liter of erythrocytes (mean corpuscular hemoglobin concentration, MCHC); total number of white blood cell count (WBC) and differential analysis including neutrophils (Neu), lymphocytes (Lym), monocytes (Mon), eosinophils (Eos) and basophils (Bas); and platelet count (PLT).

### 2.2. Analysis of Bisphenol A, Bisphenol S, Triclosan, and Resorcinol in Urine

Commercially available standards of analytical purity were used for the analysis: BPA (≥98.5%), BPS (99.9%), and RCO (99.8%) manufactured by Dr. Ehrenstorfer (Augsburg, Germany), as well as TCS (99.8%) manufactured by Sigma-Aldrich (St. Louis, MO, USA). The internal standard was BPA-d16 (Dr. Ehrenstorfer, Augsburg, Germany). The BPA, BPS, TCS, and RCO were analyzed in the first morning urine samples collected in 50 mL sterile polystyrene containers and stored at −20 °C until analysis. Prior to extraction, all laboratory glassware were thoroughly washed with acetone and n-hexane. A saline solution was used as a procedural blank, stored and processed under the same conditions as study samples. The extraction and analysis of analytes from urine was carried out using the modified method by Milić et al. [34]. A urine aliquot (3 mL) was mixed with 50 μL internal standard solution in methanol (0.2 μg/mL) and buffered with ammonium-acetate. After the hydrolyzation step for 1 h at 37 °C, the sample was preconcentrated using Chromabond^®^ XTR SPE cartridges (Macherey-Nagel, Düren, Germany; 3000 mg sorbent/3 mL volume). Elution was performed with dichloromethane (HPLC grade, stabilized with amylen; Fischer Chemical, Loughborough, UK). The eluates were dried at 35 °C under nitrogen stream pressure 1–2 bar. Phenols were then derivatized using 25 μL of N,O-bis(trimethylsilyl)trifluoroacetamide (BSTFA; PanReac AppliChem^®^, Darmstadt, Germany). Quantification of BPA, BPS, TCS, and RCO in urine samples was performed using the gas chromatography-mass spectrometry (GC-MS) method, using an Agilent Technologies GC 7890A instrument coupled with a MSD 5975C mass detector (Agilent Technologies, Santa Clara, CA, USA). Analyte separation was performed on a DB-5MS capillary column (30 m long, 0.25 mm inner diameter, 0.25 μm film thickness; manufactured by Agilent J&W, Santa Clara, CA, USA), with helium (He) as the carrier gas at a constant flow rate of 1.0916 mL/min. Injection was performed in splitless mode with a sample volume of 1 μL. The oven was heated to 50 °C for 1 min and then to 130 °C with the 20 °C/min rate (holding for 1 min). Finally, the temperature was raised to 280 °C with the 8 °C/min rate and was kept constant for 6.25 min. The total runtime was 31.75 min. Detection was carried out in electron ionization (EI) mode, at an ion source temperature of 230 °C and a quadrupole temperature of 150 °C. The MS system was set in selective ion monitoring (SIM) mode, and quantification was based on peak area using one target and two qualifier ions [34]. Complete SIM parameters, retention times, and quantification limits (LOQ) could be found in Table S1. Only concentrations greater than or equal to the LOQ were considered positive for the statistical analyses, while those below the LOQ were treated as non-detected.

### 2.3. Statistical Analysis

Descriptive statistic methods, statistical hypothesis-testing methods and methods for analyzing the dependence between variables were used for data processing. From the descriptive methods, measures of central tendency (arithmetic mean, median) and measures of dispersion (standard deviation, SD) were applied. Depending on the data distribution, parametric tests (Student’s *t*-test and analysis of variance, ANOVA) and corresponding non-parametric tests (Mann–Whitney test and Kruskal–Wallis test) were used to test statistical hypotheses. Pearson’s correlation coefficient and linear regression analysis were used as methods for analyzing dependence. Statistical hypotheses were tested at a significance level of 0.05. Odds ratios were used to compare the relative odds of the occurrence of the type of lung cancer, given exposure to the EDCs in the total analyzed group and among genders. The Kaplan–Meier survival analysis was applied for the estimation of EDCs effects on survival rate. Benjamini–Hochberg False Discovery Rate (FDR) was applied to adjust *p* values in multiple hypothesis tests to avoid type I errors (false positives) and obtained results were presented as *q*-values. Statistical analysis was performed using SPSS Statistics for Windows version 25 (IBM Corporation, Armonk, NY, USA). 

## 3. Results

A total of 190 patients (105 males and 85 females) with histopathologically confirmed advanced-stage (IIIB/IV) lung cancer were included in this study. The main characteristics of the involved patients are summarized in Table 1.

### 3.1. Frequency of Quantification of the Analyzed Phenols

**Figure 1:**
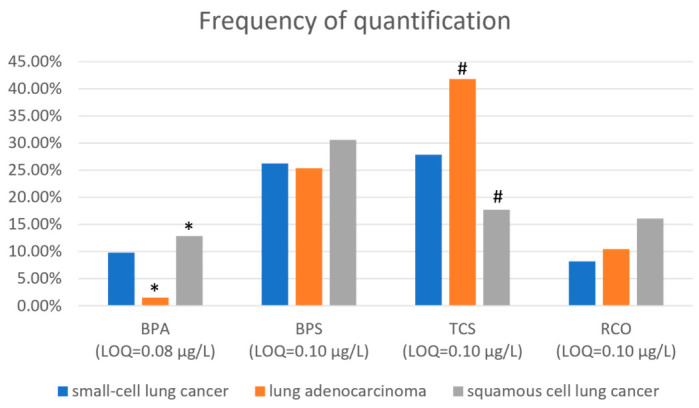
Frequency of quantification of the observed EDCs per lung cancer subtype. An asterisk is added to the *p* values below 0.05 and hash sign to *q* values below 0.05.

In the study cohort, 61 patients had small-cell lung cancer (SCLC; 45.91% female) while 129 patients had diagnosed non-small lung cancer (NSCLC), including 67 with lung adenocarcinoma (40.30% female) and 62 with squamous-cell lung cancer (48.39% female). TCS was the most frequently quantified EDC, measured in 29.47% (56/190) of urine samples, followed by BPS in 27.37% (52/190) and RCO in 11.58% (22/190), while BPA was quantified in 7.89% (15/190) of urine samples (Table 2, Figure S1). Quantification frequencies across histological lung cancer subtypes are presented in Figure 1.

**Figure 2:**
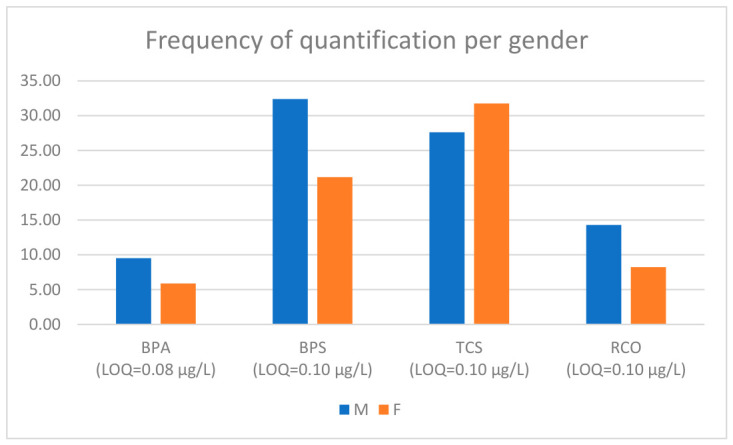
Frequency of quantification of the analyzed EDCs per gender.

Patients with lung adenocarcinoma had 3.33-fold higher odds of having quantified urinary TCS compared with patients with squamous-cell lung cancer (41.79% vs. 17.74%; OR = 3.33, 95% CI: 1.48–7.50, *p* = 0.0037, *q* = 0.0148, Figure 1). On the other hand, BPA was more frequently measured in patients with squamous-cell lung cancer than in those suffering lung adenocarcinoma (12.90% vs. 1.49%; OR = 9.78, 95% CI: 1.19–80.63, *p* = 0.0342, Figure 1). Urinary concentrations differed significantly only for RCO between patients with SCLC and those with squamous-cell lung cancer (Table 3). No statistically significant differences in quantification frequencies were observed between male and female patients for any of the four analyzed EDCs (Figure 2). However, urinary concentrations of BPA (μg/L; *p* = 0.039) and creatinine-adjusted BPS (μg/gCr; *p* = 0.043), respectively, were significantly higher in female patients than in male patients (Table 4).

At the end of follow-up (January 2026), 5.26% (10/190) of patients remained alive. Kaplan–Meier survival analysis showed no statistically significant differences in survival between patients with urinary EDCs levels (BPA, BPS, TCS and RCO, respectively) above and below the LOQ for BPA (*p* = 0.067), BPS (*p* = 0.397), TCS (*p* = 0.762) or RCO (*p* = 0.539). However, survival differences between patients with urinary BPA concentrations below and above LOQ approached statistical significance (*p* = 0.067), suggesting a trend toward lower survival among patients with measurable BPA. Given that only 15 patients had BPA above LOQ in the urine samples, these values should be interpreted cautiously. Stratified survival analyses by sex also showed no statistically significant differences between patients with quantified and non-quantified EDC levels (RCO: *p* = 0.490 in males and *p* = 0.866 in females; TCS: *p* = 0.639 in males and *p* = 0.990 in females; BPA: *p* = 0.307 in males and *p* = 0.121 in females; BPS: *p* = 0.112 in males and *p* = 0.704 in females).

### 3.2. Cardiometabolic Parameters and Urinary Phenols

The cardiometabolic risk factors in this study included obesity parameters (BMI, waist and hip circumference, WHR, WtHR), systolic and diastolic blood pressure, pulse rate, serum glucose levels, and serum CK and CK-MB fraction. In the total sample, diastolic blood pressure was higher in patients with quantified BPA than in those with BPA below LOQ, with marginal significance (*p* = 0.092, Table S2), as well as in the male subgroup (*p* = 0.096). In addition, in the male subgroup, diastolic BP was associated with urinary BPA levels (*p* = 0.042, Table S3). In female patients with urinary BPA above LOQ, waist-to-height ratio was significantly higher (*p* = 0.021) in comparison to those with urinary BPA levels below LOQ. In contrast, waist circumference (*p* = 0.060) and hip circumference (*p* = 0.059) showed marginally significant differences (Table S2).

Regarding BPS exposure, only marginal differences were observed in the waist-to- height ratio in the male cohort (*p* = 0.065) and in the waist-to-hip ratio (*p* = 0.064) in the female cohort. In the total sample, waist circumference, diastolic blood pressure, and pulse rate were associated with urinary BPS (*p* = 0.056, *p* = 0.036, and *p* = 0.073, respectively). Patients with urinary BPS concentrations above the LOQ had higher serum CK values (*p* = 0.030) compared to those below the LOQ (Table S4). Among male patients (Table S4), CK was statistically higher in those with measurable urinary BPS (*p* = 0.008). Additionally, regression analysis revealed an association between CK-MB and urinary BPS among males (*p* = 0.001, *q* = 0.020, Table S5).

TCS exposure resulted in higher WC and diastolic BP in patients with quantifiable pollutant but borderline significant (*p* = 0.094 and *p* = 0.084, respectively, Table S6). In male patients, a higher waist-to-hip ratio, diastolic BP, and pulse rate were observed (*p* = 0.063–0.094, Table S6). The urinary TCS levels were associated with pulse rate (*p* = 0.046) in the total sample, as well as with CK-MB in female patients (*p* = 0.053, Table S7).

In addition, a higher pulse rate was recorded in male patients with measured RCO in urine (*p* = 0.097, Table S8). In the overall cohort, the waist-to-hip ratio was associated with urinary RCO levels (*p* = 0.064, Table S9). In the male cohort, waist circumference and the waist-to-height ratio were also associated with RCO (*p* = 0.075 and *p* = 0.083, respectively), while in the female cohort, RCO was only associated with waist circumference (*p* = 0.014, Table S9).

**Figure 3:**
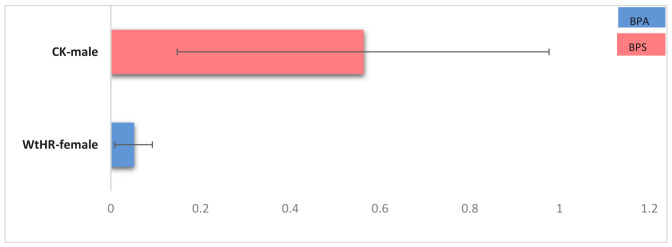
Statistically significant mean differences (95% confidence intervals) for cardiometabolic parameters in patients with urinary BPA and BPS above and below LOQ in each sex cohort.

Figure 3 summarizes the mean differences (95% confidence intervals) in cardiometabolic parameters in patients with urinary BPA or BPS levels above and below LOQ in female and male patients (*p* < 0.05). None of the parameters had an FDR-adjusted *p*-value (*q*-value) below 0.05.

### 3.3. Liver, Kidney Function and Inflammatory Parameters and Urinary Phenols

Liver (ALT, AST, GGT, LDH, ALP, total and direct bilirubin) and kidney (urea, serum creatinine, uric acid) function parameters were analyzed. Positive associations were noticed between BPA levels and ALP (*p* = 0.021, Table S3), as well as uric acid (*p* = 0.088) in the total cohort. Kidney function parameters, including urea (*p* = 0.004, *q* = 0.041), serum creatinine (*p* = 0.002, *q* = 0.027), and uric acid (*p* = 0.001, *q* = 0.041), were higher in female patients with quantifiable urinary BPA (Table S2). In the female cohort, urea values were also associated with BPA urinary concentrations (*p* = 0.008, Table S3). In the male subgroup, serum creatinine (*p* = 0.030) and uric acid (*p* = 0.032) concentrations were lower in patients with urinary BPA concentrations above the LOQ than in those with concentrations below the LOQ. Moreover, urinary BPA levels (μg/g Cr) were inversely associated with serum creatinine (*p* = 0.024, Table S3) in male patients and showed a marginal inverse association with uric acid (*p* = 0.057) after adjustment for age and BMI. Finally, ALP values were lower in male patients with quantified BPA with marginal significance (*p* = 0.071, Table S2).

Serum creatinine (*p* = 0.029) and uric acid (*p* = 0.019) concentrations were also higher in patients with quantifiable urinary BPS (Table S4). Among male patients (Table S4), uric acid (*p* = 0.023) and AST (*p* = 0.096) were higher in those with measurable urinary BPS. Uric acid concentrations were also associated with urinary BPS in the total sample (*p* = 0.015), as well as in the male cohort (*p* = 0.050, *q* = 0.065, Table S5). Also, urinary BPS levels were positively associated with serum GGT (*p* = 0.004) and LDH (*p* = 0.040), particularly among male patients (*p* = 0.001, *q* = 0.020, and *p* = 0.007, *q* = 0.068, respectively).

Although no statistically significant differences were observed between patients with TCS above and below LOQ (Table S6) regarding liver and kidney function, urinary TCS concentrations were associated with direct bilirubin (*p* = 0.022), uric acid (*p* = 0.008 for μg/L and *p* = 0.045 for μg/gCr), and LDH (*p* = 0.065) in female patients (Table S7).

Among the female cohort, total bilirubin and uric acid concentrations were lower in patients with urinary RCO levels above the LOQ than in those below the LOQ (*p* = 0.078 and *p* = 0.091, respectively, Table S8). After adjustment for age and BMI, urinary RCO levels were marginally associated with AST serum levels in the overall cohort (*p* = 0.060 for μg/L; *p* = 0.086 for μg/gCr), while significant associations were observed among male patients (*p* = 0.010 for μg/L and *p* = 0.013 for μg/gCr, respectively) (Table S8). Moreover, RCO was marginally associated with urea and ALT in the male subgroup (*p* = 0.078 and *p* = 0.090, respectively), while RCO was associated with serum creatinine in the female subgroup (*p* = 0.072, Table S9).

**Figure 4:**
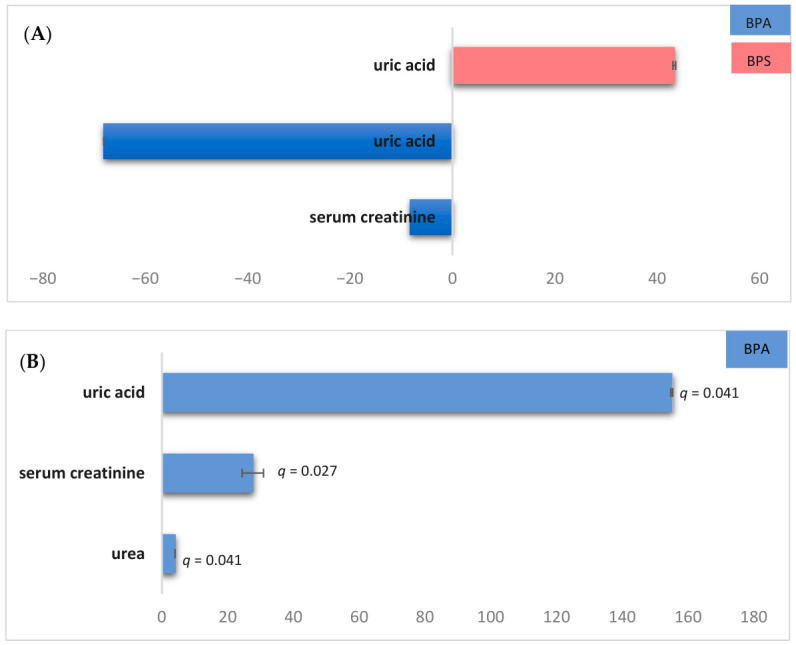
Statistically significant (*p* < 0.05) mean differences (95% confidence intervals) for liver and kidney biomarkers in patients with urinary BPA and BPS levels, respectively, above and below LOQ in (**A**) male and (**B**) female subgroups. Significant FDR *q*-values for specific biomarkers are given in the figure.

**Figure 5:**
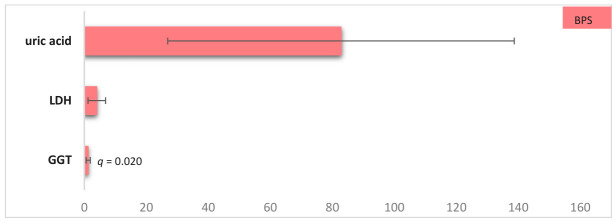
β-values (95% confidence intervals) for liver and kidney biomarkers significantly associated (*p* < 0.05) with BPS after age- and BMI-adjustments in male patients. Significant FDR *q*-values for specific biomarkers are given in the figure.

Figure 4 presents statistically significant (*p* < 0.05) mean differences (95% confidence intervals) for liver and kidney biomarkers between patients with and without measurable urinary BPA and BPS concentrations in both genders. The significant associations of liver and kidney biomarkers with urinary BPS concentrations after age and BMI adjustments in male patients are given in Figure 5.

### 3.4. Parameters of Hematological Status and Immune Response and Urinary Phenols

Hematological and immune parameters included complete blood count with leukocyte differential (RBC, Hgb, Hct, MCV, MCH, MCHC, WBC, neutrophils, lymphocytes, monocytes, eosinophils, basophils), platelet count, and CRP. In the total sample, urinary BPA levels were associated with basophils and platelets (*p* = 0.032 and *p* = 0.038, respectively). Among male patients, basophil counts were lower (*p* = 0.011), whereas mean corpuscular hemoglobin concentration (MCHC) values were higher (*p* = 0.004) in those with quantifiable urinary BPA. CRP values were lower in males with BPA above the LOQ (*p* = 0.024, Table S2) and negatively associated with BPA levels (*p* = 0.009, Table S3). In the same subgroup, platelets were associated with BPA concentrations (*p* = 0.009). On the other hand, higher neutrophil levels (*p* = 0.002, *q* = 0.027) and monocyte counts (*p* = 0.044) were observed, followed by higher red blood cell counts (*p* = 0.056) in BPA-positive female patients, as given in Table S2. In addition, eosinophils in total count and percentage were associated with BPA levels in females (*p* = 0.040 and *p* = 0.049, respectively, Table S3).

Patients with urinary BPS concentrations above the LOQ had higher red blood cell count (*p* = 0.014) in comparison with those below the LOQ (Table S4). Marginally significant differences were observed for neutrophils (*p* = 0.062) and monocytes (*p* = 0.072), where some associations were sex-specific. Among male patients (Table S4), red blood cell count (*p* = 0.045) was statistically higher in those with measurable urinary BPS, whereas monocyte count was elevated in BPS-positive female patients (*p* = 0.043). Regression analysis adjusted for age and BMI demonstrated an association between urinary BPS concentration and monocyte percentage (*p* = 0.003, *q* = 0.059) and basophil count (*p* = 0.001, *q* = 0.039) in female patients. In male patients, urinary BPS levels were associated only with basophils (*p* = 0.022) (Table S5).

Among patients with urinary TCS concentration above the LOQ, monocyte counts were statistically significantly lower (*p* = 0.036), while CRP levels showed a marginally significant decrease (*p* = 0.052), compared with those with concentrations below the LOQ (Table S6). Among male patients, total leukocyte counts were significantly lower in TCS-positive individuals (*p* = 0.007), with similar trends observed across leucocyte subtypes, including monocytes (*p* = 0.020), lymphocytes (*p* = 0.021), eosinophils (*p* = 0.032), neutrophils (*p* = 0.027), and basophils (*p* = 0.091) (Table S6). Among female patients, only the percentage of monocytes differed significantly (*p* = 0.013, Table S6). Regression analysis demonstrated associations between urinary TCS concentrations and WBC (*p* = 0.047) and lymphocyte counts (*p* = 0.036) in female patients (Table S7). One interesting trend across the entire cohort and in the male subgroup was that urinary TCS levels were positively associated with hemoglobin (*p* = 0.043 and *p* = 0.055, respectively) and hematocrit (*p* = 0.034 and *p* = 0.057, respectively) (Table S7).

Patients with quantifiable urinary RCO had marginally higher monocyte counts in the overall cohort and in the male subgroup (*p* = 0.073 and *p* = 0.051, respectively) compared with those with RCO concentrations below the LOQ (Table S8). Other differences were not statistically significant, although lower CRP concentrations were observed in male patients with quantifiable RCO (*p* = 0.093). Regression analysis indicated a weak association between lymphocyte count and RCO levels (*p* = 0.067) in the total sample, and with neutrophil, lymphocyte, monocyte and basophil percentages in the female subgroup (*p* = 0.061–0.095, Table S9).

**Figure 6:**
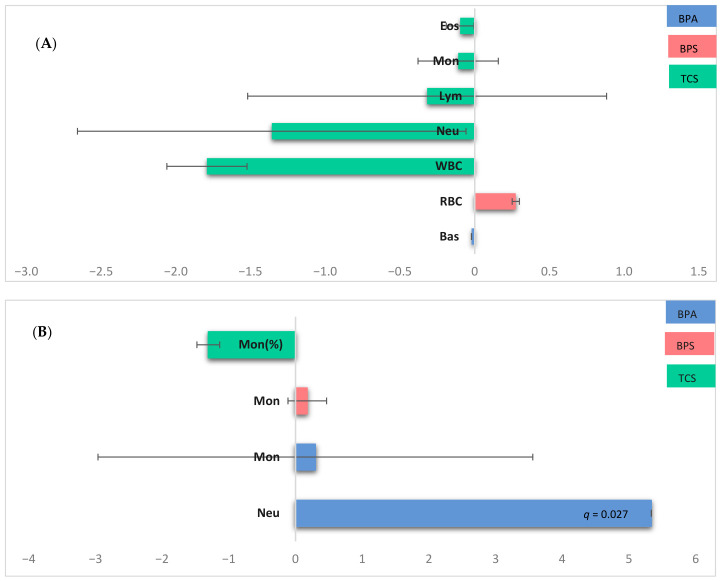
Statistically significant (*p* < 0.05) mean differences (95% confidence intervals) for blood cell count and % between patients with and without measurable urinary BPA, BPS and TCS concentrations in (**A**) males and (**B**) females. Significant FDR *q*-values for specific biomarkers are given within Figure.

Figure 6 summarizes the statistically significant (*p* < 0.05) mean differences (95% confidence intervals) for blood cell count and % between patients with and without measurable urinary BPA, BPS and TCS concentrations for each sex.

## 4. Discussion

The etiopathogenesis of lung cancer is increasingly recognized as involving not only classical carcinogenic exposures such as tobacco smoke and air pollution but also endocrine and metabolic dysregulation. In particular, emerging evidence suggests a role of estrogen signaling and hormone-dependent pathways in lung tumor development and progression [35]. EDCs have been extensively investigated in hormone-dependent malignancies such as breast and prostate cancer [36]. However, data related to their presence and possible importance in the pathogenesis of lung cancer are scarce. Because several phenolic EDCs interact with nuclear hormone receptors expressed in lung tissue and may influence xenobiotic metabolism, oxidative stress responses, and inflammatory signaling pathways, biomonitoring studies in oncology populations provide an important opportunity to investigate their potential role in cancer-related biological processes. In this context, the present study represents one of the first assessments of urinary exposure to selected phenolic EDCs in patients with advanced lung cancer in Serbia and contributes to the growing body of evidence linking environmental exposures with cancer-associated metabolic and immunological alterations.

### 4.1. Frequency of Quantification, Concentrations of Analyzed Compounds, and Survival Rate

In the present study, triclosan (TCS), bisphenol S (BPS), bisphenol A (BPA), and resorcinol (RCO) were quantified in a subset of patients with advanced lung cancer, with TCS representing the most frequently quantified compound. Because population-level biomonitoring data for these compounds are currently unavailable in Serbia, the present findings provide one of the first reference datasets describing exposure patterns in oncology patients within this region. Compared with large biomonitoring programs such as NHANES [37] and HBM4EU [38], quantification frequencies of BPA and BPS observed in this cohort were substantially lower. In NHANES, BPA detection frequencies exceeded 90% [37], whereas European HBM4EU data reported BPA detection in more than 92% of samples and BPS in approximately two-thirds of participants [38]. These discrepancies may reflect differences in sampling period, demographic structure, exposure sources, analytical sensitivity, and health status of the study population. Importantly, patients with advanced malignancies often exhibit altered metabolism, modified dietary behavior, and reduced exposure to consumer products, which may contribute to lower urinary quantification frequencies compared with the general population. Despite lower quantification frequencies, mean urinary concentrations of BPA and BPS in the present cohort exceeded recently proposed health-based guidance values reported by the European Environment Agency (EEA), suggesting that exposure levels in this patient population remain environmentally relevant. This finding is particularly important given the increasing regulatory replacement of BPA with structural analogues such as BPS, which are now recognized as biologically active substitutes rather than safer alternatives [39]. The EEA has also adopted an exposure threshold for urinary BPA of 11.5 ng/L (0.0115 μg/L) as a health-acceptable limit. Comparing this value with the results obtained in our study, it is observed that the mean concentrations of BPA (0.1627 μg/L) and BPS (0.3402 μg/L) recorded in samples of patients with lung cancer are several times higher. These results indicate that exposure to bisphenols in the observed population exceeds currently established safety thresholds and may represent an additional health risk. Consistent with this interpretation, the quantification frequency of BPS exceeded that of BPA in the present cohort, supporting the hypothesis that substitution trends in consumer materials are already reflected in human biomonitoring data. Environmental monitoring studies conducted in Serbia have also identified bisphenols in surface and wastewater systems, suggesting that environmental contamination represents a plausible exposure source at the population level.

In a study conducted by Qu et al. [40] on a population of lung cancer patients in China, higher quantification frequencies of BPA (95%) and BPS (53%) were noted compared to the results of our study. Also, the mean concentrations of BPA were higher (0.95 μg/gCr), while the values for BPS (0.63 μg/gCr) were comparable to the results of our sample. The higher quantification frequency of BPS compared to BPA is consistent with the increasing use of BPS as a substitute for BPA. According to available studies, some of the confirmed sources of exposure to bisphenols in the territory of our country include polluted surface and waste waters, with possible seasonal variations in concentrations [41,42]. Although a cross-sectional study such as this cannot confirm causality, the results of a case–control study by Qu et al. [40] suggest a possible causal relationship between bisphenol exposure and lung cancer risk, indicating that elevated concentrations of BPA in urine are significantly positively correlated with the risk of lung cancer (*p* < 0.05).

Compared to global data on TCS exposure, where it was detected in more than 75% of urine [43], the obtained results suggest a lower exposure in the studied population. However, the relatively higher quantification rate of TCS compared with BPA in the present study suggests that exposure pathways remain active and may be underestimated in environmental exposure assessments.

In contrast to bisphenols and triclosan, population-level biomonitoring data for RCO remain scarce, limiting direct comparison with international datasets [44]. Relatively low quantification frequencies observed in this study may be due to limits of quantification in the range of 0.08 to 0.10 μg/L, which may lead to an underestimation of actual exposure. Although concentrations below the LOQ do not allow for precise measurement, their presence may indicate chronic low-dose exposure, which may have biological significance in the context of endocrine disruption. Additionally, given that these are oncology patients at an advanced stage of the disease, it is possible that product use patterns, fluid intake, and metabolic characteristics do not reflect typical exposure values of the general population, but represent a potentially specific risk profile of this population. Changes in metabolism in patients with malignant diseases may contribute to the accelerated elimination of these compounds.

Sex-specific exposure patterns observed in this cohort were consistent with previously reported differences in lifestyle-related exposure pathways. TCS was quantified with a higher frequency in the female population. Although quantification frequencies were broadly comparable between men and women, urinary concentrations normalized to creatinine were generally higher in female patients, reaching statistical significance for BPS (Table 4). In addition, statistically significantly higher concentrations in women were observed for BPA expressed in μg/L. These findings may reflect sex-specific exposure sources, including personal care product use and differences in endocrine regulation and xenobiotic metabolism [45]. Although diet, food storage habits, and personal hygiene routines are beyond the scope of this study, they may have a significant impact on individual and gender differences in exposure to these compounds. However, gender related differences should be interpreted with caution due to the relatively low frequency of quantification, particularly for BPA and RCO.

In addition to differences in exposure, gender differences in the specificities of lung cancer are known. Molecular alterations, such as increased frequency of EGFR mutations and increased expression of KRAS (Kirsten rat sarcoma viral oncogene homologue), are more prevalent in women with lung adenocarcinoma, and may influence interactions between environmental exposures and tumor biology. Increased sensitivity to air pollution and passive smoking exposure has also been reported in female populations and may contribute to sex-dependent exposure–response relationships involving EDCs [35].

The borderline significance of BPA with survival rate despite the small sample size is in accordance with previously reported BPA association with cancer mortality, especially when patients were exposed to low doses [46]. The observed association between urinary BPA quantification and reduced survival probability approached statistical significance despite the limited number of BPA-positive cases. Although this finding requires cautious interpretation, it is consistent with previous epidemiological studies reporting associations between low-dose BPA exposure and increased cancer-related mortality. No survival associations were observed for the remaining compounds, suggesting either weaker biological relevance or insufficient statistical power to detect moderate exposure effects.

### 4.2. Cardiometabolic Parameters

Alterations in cardiometabolic biomarkers observed in this cohort provide additional evidence supporting the potential association between EDCs and systemic effects in patients with advanced lung cancer. Increasingly, cardiometabolic dysfunction is recognized not only as a comorbidity but also as a biologically relevant component of cancer progression, influencing inflammation, oxidative stress, immune regulation, and treatment response [47,48]. Furthermore, a multicenter study that included over 300,000 subjects showed an association between components of the metabolic syndrome and lung cancer, highlighting their importance as a risk factor for this malignancy [49]. Attention is also drawn to the concept of metabolic variability, which is characterized by fluctuations in body mass, blood glucose, systolic blood pressure, and total cholesterol, which, regardless of average values, have been identified in several studies as risk factors for malignancy, including lung cancer. In some studies, these relationships are observed predominantly in men, which can be explained by differences in histological tumor subtypes and metabolic response [47].

Although obesity is considered a major risk factor for several common types of cancer, in the case of lung cancer, a high BMI is paradoxically associated with a reduced risk, especially in smokers. Contrary to data suggesting a link between lung cancer and BMI, significantly fewer studies included measures of central obesity, which is a key predictor of adverse health outcomes associated with high body fat. WHR and WtHR are associated with an increased risk of lung cancer, regardless of gender and smoking status, and the association was particularly prominent in the case of squamous-cell carcinoma [50,51,52].

A heterogeneous group of patients with lung cancer was included in this study, with BMI varying from 14.20, which indicates marked malnutrition characteristic of cachectic patients in the terminal stages of the disease, to 40.43, the obesity domain. The mean value of BMI was 24.88, a value at the upper limit of normal nutrition. All regression analyses were adjusted for BMI and age. Notably, female patients with urinary BPA concentrations above the LOQ exhibited higher WtHR compared with those below the LOQ (Figure 3). According to World Health Organization criteria, the mean waist circumference (91.29 cm) and WHR (0.93) indicate a higher category of central obesity [52].

A trend toward associations between the analyzed compounds and cardiometabolic parameters (diastolic blood pressure, pulse, CK, and CK-MB) was observed, although mostly not statistically significant. Participants with BPS ≥ LOQ had significantly higher CK levels, with a stronger association in men (Figure 3). CK-MB also showed a positive association with BPS concentrations (μg/L) in males that remained significant after Benjamini–Hochberg FDR adjustment (*q* = 0.020). Moreover, diastolic BP was associated with urinary BPS concentrations in the total cohort and with urinary BPA concentrations in the male subgroup, whereas pulse rate was associated with urinary TCS concentrations. Literature reports support the association between EDCs and cardiovascular parameters, including increased blood pressure and heart rate [53,54,55].

Although no clear statistically significant hypertensive effect after Benjamini–Hochberg FDR adjustment was observed, the results are consistent with evidence linking EDCs to subclinical cardiovascular dysregulation and increased vascular vulnerability at diagnosis. Mechanistically, BPA promotes endothelial oxidative stress, leading to dysfunction and inflammation, while data on newer bisphenols remain limited but suggest similar pro-oxidative effects [56]. Cardiac rhythm disturbances, including tachycardia, are also common in lung cancer patients, often as a consequence of therapy [57]. Since this study included treatment-naïve patients, therapy-related effects are unlikely, but the findings may have implications for future treatment.

The trend toward higher diastolic pressure and heart rate in men is consistent with known sex differences in cardiovascular risk, partly due to the protective vascular effects of estrogen in women [58]. Rubinstein et al. [59] reported that higher urinary levels of phenolic EDCs (including BPA, BPF, TCS, and TCC) are associated with sex-specific alterations in cardiac electrical activity, which are clinically relevant and may indicate increased susceptibility to cardiotoxic effects in certain subpopulations. CK and CK-MB are classical markers of acute myocardial infarction, but their levels—particularly the CK-MB/CK ratio—may also be elevated in malignant diseases independent of cardiac injury [60,61]. Blood glucose ranged from 3.80 to 28.20 mmol/L (mean 6.71 mmol/L), indicating a tendency toward hyperglycemia; however, no significant association was found between glycemia and the analyzed EDCs.

Older age is a known risk factor for lung cancer, reflecting cumulative exposure to environmental toxins and a higher burden of comorbidities [62]. In this study, no overall association between age and urinary levels of the analyzed compounds was observed. The cardiometabolic alterations observed in this study support the hypothesis that exposure to phenolic EDCs may contribute to systemic metabolic variability in patients with advanced lung cancer. Because cardiometabolic biomarkers represent integrative indicators of inflammation, organ function, and endocrine signaling, their association with urinary EDC levels strengthens the biological plausibility of environmental exposure contributing to cancer-related physiological heterogeneity.

### 4.3. Liver, Kidney Function and Inflammatory Parameters

Given the central role of the liver and kidneys in EDC metabolism and elimination, their functional variability may influence EDC distribution and clearance in patients with advanced lung cancer, reflecting systemic inflammation, organ burden, and the altered capacity for metabolism and elimination [63]. In this context, several statistically significant and borderline associations were observed between EDCs levels and these parameters, suggesting potential interactions relevant for pharmacokinetics and treatment tolerability.

For BPA, positive associations were observed with ALP remaining independent of BMI and age. Uric acid, urea and serum creatinine were statistically significantly higher in female patients with measurable BPA (*q* = 0.041, *q* = 0.041 and *q* = 0.027, respectively, Figure 4). Given the small sample size of female patients with BPA concentration above the LOQ, these data should be interpreted with caution. Similar findings were reported by Sladič et al. [64], who noted lower BPA levels compared to HBM4EU data but a positive correlation between urinary BPA and creatinine, suggesting a potential link with renal impairment in oncology patients.

Numerous studies in general populations (South Korea, USA, China, Pakistan, and Italy) have shown significant associations between urinary BPA levels and liver dysfunction markers, including elevated ALT, AST, GGT, LDH, and ALP. Positive correlations with total bilirubin and negative correlations with serum proteins and albumin have also been reported. These associations are particularly evident in individuals with metabolic disorders and hepatocellular carcinoma, supporting the hepatotoxic potential of BPA in both general and vulnerable populations [65].

For BPS, higher uric acid levels were associated with higher concentrations of this EDC (*q* = 0.065) in the male cohort, indicating independence from confounders. BPS levels were also positively associated with GGT (*q* = 0.020, Figure 5), and with LDH (*q* = 0.068) in the male subgroup, independent of BMI and age. Although evidence for BPS is still more limited compared to BPA, animal studies and emerging human data on BPA analogues suggest potential effects on liver enzymes (ALT, AST, GGT) and hepatocellular injury markers, supporting possible hepatotoxic mechanisms [65,66,67,68]. Despite the lack of evidence regarding TCS exposure and kidney function, in this study uric acid in female patients was associated with urinary TCS levels. Uric acid, the final product of purine metabolism, is influenced by purine catabolism and renal transport mechanisms, and its dysregulation is linked to gout, renal dysfunction, obesity, metabolic syndrome, diabetes, and inflammatory states. Both hypo- and hyperuricemia have been associated with increased cancer risk and a U-shaped relationship with overall mortality, including oncological outcomes [69]. Exposure to BPA and its analogues, including BPS, has been associated in population studies with serum uric acid levels and hyperuricemia, often showing a non-linear (inverted U-shaped) dose–response relationship, where moderate exposure is most strongly associated with increased risk [70].

### 4.4. Parameters of Hematological Status and Immune Response

The most notable associations with urinary EDCs were observed in this group of studied parameters. In erythrocyte parameters, TCS showed positive association with hemoglobin and hematocrit, which remained after adjustment for BMI and age. For BPA, higher MCHC was observed in the male population above the LOQ. In the case of BPS, significantly higher RBC counts were found in the total sample and in men. A Korean study in pregnant women also reported stronger effects of BPS compared to BPA on RBC, hemoglobin, and hematocrit, although with inverse (negative) associations [71]. While not directly comparable due to population differences, pregnancy also represents a state of increased susceptibility to hematological alterations. In oncology patients, anemia is highly prevalent (30–90%), arising from both tumor-related mechanisms and therapy effects [72]. Similar effects of TCS have been reported in aquatic experimental models, where reductions in RBC, hemoglobin, and related hematological parameters were observed [73]. However, the lack of comparable clinical studies highlights the need for further research.

For BPA, a statistically significant positive association with platelet count was observed, independent of BMI and age. Although platelets play a central role in hemostasis, the effects of BPA and its analogues on coagulation remain insufficiently characterized. Experimental data indicate that BPA and BPS do not directly affect platelet aggregation induced by arachidonic acid, but at higher doses may prolong fibrin clot formation, suggesting potential effects on coagulation factors and disruption of hemostatic balance [74].

Regarding immune response parameters, significantly lower leukocyte subpopulations were recorded for TCS in the total sample (monocytes,) and in men (WBC, neutrophils, lymphocytes, monocytes, eosinophils; Figure 6A), while in women only monocyte percentage was significantly reduced (Figure 6B). For BPS, higher monocyte counts were observed in women, while in the total sample decreasing trends were noted for neutrophils. BPS in females was also associated with monocyte percentage (*q* = 0.059) and basophil count (*q* = 0.039), independent of BMI and age. For BPA, lower basophil values were observed in men, with a positive association with basophils in the total sample after adjustment for BMI and age. BPA was associated with significantly lower CRP in men, suggesting potential modulation of systemic inflammatory status. Finally, neutrophils were higher in the women subgroup with quantified BPA (*q* = 0.027, Figure 6B).

Parameters of immune response and systemic inflammation—including leukocyte count and differential, CRP, and hemostatic biomarkers—are commonly evaluated together to assess immune homeostasis in malignant diseases. Their balance and individual alterations reflect interactions between the tumor microenvironment, systemic inflammation, and hematopoietic response, and may have prognostic value in cancer patients [75]. Some studies have shown that systemic inflammatory markers are associated with poor outcomes in advanced inoperable cancers [76]. Elevated pre-therapeutic values of hemoglobin, monocytes, lymphocytes, CRP, neutrophil-to-lymphocyte ratio, lymphocyte-to-monocyte ratio, and platelet-related indices have been linked to shorter survival and faster disease progression in lung cancer, independent of clinical risk factors [77]. These parameters may also contribute to resistance to chemotherapy and immunotherapy, with the neutrophil-to-lymphocyte ratio being the most frequently studied indicator [77,78,79]. Immunotherapy has significantly improved outcomes in lung cancer, but is also associated with immune-mediated adverse effects. In this context, hematological parameters such as neutrophil-to-lymphocyte ratio and absolute counts of eosinophils, monocytes, and lymphocytes are increasingly recognized as potential predictive biomarkers for both treatment response and safety profile [80,81].

### 4.5. Strengths and Limitations

This is a pioneer study conducted only on patients with histopathologically confirmed different subtypes of advanced lung cancer that simultaneously assessed urinary levels of four EDCs (BPA, BPS, TCS, and RCO). The major strength is that comprehensive laboratory findings were associated with different EDCs urinary levels because EDCs affect multiple molecular and physiological pathways rather than a single target. This approach provides a more integrated evaluation of EDC exposure effects on human health than focusing on a limited set of endpoints. The analyses adjusted for age and BMI identified significant associations between the investigated variables. The findings highlight the importance of considering both the original adjusted analyses and the results after correction for multiple comparisons. While some associations did not withstand FDR correction, the observed effect patterns may provide valuable insights and generate hypotheses for future studies with larger sample sizes and greater statistical power.

Owing to the cross-sectional design of the present study and the limited number of patients with quantified levels for some compounds, these findings should be interpreted as hypothesis-generating and require confirmation in prospective exposure–outcome studies integrating longitudinal biomonitoring approaches. Urinary concentrations of BPA, BPS, TCS and RCO were quantified in a single spot morning urine sample, which may not accurately reflect chronic exposure due to relatively short half-lives of these compounds. Furthermore, the low frequencies of quantification, particularly in the sex-stratified analyses, represent a major limitation of this study. Consequently, these subgroup analyses and their corresponding findings should be interpreted cautiously because of the limited statistical power. Finally, the Kaplan–Meier survival analysis should be considered preliminary and requires confirmation in larger cohorts.

## 5. Conclusions

In this biomonitoring study of patients with inoperable lung cancer, TCS was the most frequently quantified compound in the first morning urine samples, followed by BPS, RCO and BPA. There are statistically significant differences in certain clinical parameters between patients of both sexes with inoperable lung cancer based on exposure to BPA, BPS, TCS and RCO, and the degree of expression of these associations differs depending on the analyzed compound and specific parameter. The most pronounced trends of association were recorded for BPA and BPS. The fact that associations were observed in models adjusted for relevant confounding factors, including age and BMI, suggests that the identified relationships may represent potentially meaningful patterns that warrant additional investigation. Further research is needed to determine the possible effects of exposure to phenolic compounds on cardiometabolic parameters, liver and kidney function, as well as the patient’s hematological and immune status.

## Figures and Tables

**Table 1:** The main characteristics of the involved patients.

Parameter	Min	Max	Mean	±SD
Age (years)	39.00	84.00	65.6684	8.5239
BMI	14.20	40.43	24.8795	4.8152
Waist circumference (cm)	58.00	130.00	91.2857	14.4828
Hip circumference (cm)	58.00	127.00	98.2912	10.8393
WHR	0.61	1.32	0.9281	0.1004
WtHR	0.36	0.81	0.5410	0.0836
BP systolic (mmHg)	90.00	190.00	125.0761	18.2573
BP diastolic (mmHg)	50.00	116.00	72.6923	10.5009
Pulse (bpm)	47.00	120.00	81.0380	14.7142
RBC (×10^12^/L)	2.83	8.60	4.4701	0.5787
WBC (×10^9^/L)	4.40	86.00	9.9929	6.6176
Neu (×10^9^/L)	2.81	32.35	6.9284	3.2448
Neu (%)	42.00	91.00	70.7554	8.8520
Lym (×10^9^/L)	0.46	4.87	1.7396	0.7107
Lym (%)	5.00	48.00	19.1312	7.8250
Mon (×10^9^/L)	0.14	2.19	0.7386	0.3019
Mon (%)	2.00	14.00	7.8296	2.3629
Eos (×10^9^/L)	0.00	2.06	0.1524	0.2283
Eos (%)	0.00	14.70	1.5827	1.9631
Bas (×10^9^/L)	0.00	0.21	0.0593	0.0350
Bas (%)	0.00	1.70	0.6303	0.3197
Hgb (g/L)	76.00	167.00	130.0691	16.3452
Hct (L/L)	0.22	0.50	0.3877	0.0458
MCV (fL)	0.92	99.00	86.8182	8.6801
MCH (pg)	20.90	34.40	29.2862	2.4617
MCHC (g/L)	310.00	368.00	335.3564	9.9235
PLT (×10^9^/L)	97.00	733.00	321.8617	114.3716
ALT (μkat/L)	0.00	3.32	0.3662	0.3708
AST (μkat/L)	0.11	1.48	0.3588	0.2154
GGT (μkat/L)	0.13	23.08	0.8857	1.9537
LDH (μkat/L)	0.87	53.00	7.7605	5.3962
ALP (μkat/L)	0.59	8.28	1.6030	0.8694
Total bilirubin (µmol/L)	2.50	23.30	7.2817	3.5274
Direct bilirubin (µmol/L)	0.40	7.60	2.5516	1.0226
Urea (mmol/L)	1.80	23.50	6.1053	2.6713
Serum creatinine (µmol/L)	34.00	162.00	72.2735	20.3606
CRP (mg/L)	0.30	216.90	35.6022	47.7665
CK (μkat/L)	0.10	5.44	1.2238	0.9363
CK-MB (μkat/L)	0.09	4.66	0.3939	0.4995
Uric acid (µmol/L)	77.00	684.00	296.4898	97.2771
Glucose (mmol/L)	3.80	28.20	6.7116	2.6135

BMI—body mass index; WHR—waist-to-hip ratio; WtHR—waist-to-height ratio; BP—blood pressure; RBC—red blood cells; WBC—white blood cells; Neu—neutrophils; Lym—lymphocytes; Mon-monocytes; Eos—eosinophils; Bas—basophils; Hgb—hemoglobin; Hct—hematocrit; MCV—mean corpuscular volume; MCH—mean corpuscular hemoglobin; MCHC—mean corpuscular hemoglobin concentration; PLT—platelets; ALT—alanine aminotransferase; AST—aspartate aminotransferase; GGT—gamma-glutamyl transferase; LDH—lactate dehydrogenase; ALP—alkaline phosphatase; CRP—C-reactive protein; CK—creatine kinase; CK-MB—creatine kinase MB.

**Table 2:** Mean values with SD, medians, minimum, maximum values as well as percentiles of urinary concentrations for BPA, BPS, TCS and RCO, respectively, given in μg/L (and in μg/gCr).

		BPA	BPS	TCS	RCO
N	≥LOQ	15	56	22	52
	<LOQ	175	134	168	138
Mean μg/L (μg/gCr)		0.1627 (0.4923)	0.3402 (0.6269)	0.1612 (0.5349)	1.5773 (2.6616)
Median μg/L (μg/gCr)		0.1000 (0.1405)	0.2000 (0.3338)	0.1000 (0.2712)	1.1000 (1.6024)
Std. Deviation μg/L (μg/gCr)		0.1161 (0.9523)	0.3905 (1.1263)	0.1414 (1.0700)	1.6579 (2.8451)
Minimum μg/L (μg/gCr)		0.10 (0.04)	0.10 (0.05)	0.10 (0.04)	0.10 (0.28)
Maximum μg/L (μg/gCr)		0.50 (3.71)	1.70 (7.43)	0.90 (7.30)	6.80 (10.42)
Percentiles μg/L (μg/gCr)	25	0.1000 (0.0746)	0.1000 (0.1289)	0.3750 (0.4643)	0.1000 (0.1502)
	75	0.2000 (0.6387)	0.2000 (0.6387)	2.1000 (4.1229)	0.4000 (0.6781)

LOQ: BPA—0.08 µg/L; BPS—0.1 µg/L; TCS—0.1 µg/L; RCO—0.1 µg/L.

**Table 3:** Comparison of the urinary concentrations of the analyzed EDCs among different cancer subtypes.

	μg/L			μg/gCr		
	Small-Cell Lung Cancer	Lung Adenocarcinoma	Squamous-Cell Lung Cancer	Small-Cell Lung Cancer	Lung Adenocarcinoma	Squamous-Cell Lung Cancer
BPA	0.133 ± 0.052	0.100	0.193 ± 0.151	0.235 ± 0.173	0.14	0.729 ± 1.286
BPS	0.306 ± 0.293	0.394 ± 0.461	0.321 ± 0.410	0.431 ± 0.307	0.649 ± 0.912	0.772 ± 1.649
TCS	0.161 ± 0.074	0.146 ± 0.157	0.200 ± 0.179	0.444 ± 0.304	0.561 ± 1.360	0.610 ± 1.065
RCO	3.020 ± 2.180 *	1.614 ± 1.721	0.830 ± 0.741 *	**4.411 ± 2.821 #**	2.735 ± 3.780	**1.736 ± 1.779 #**

* *p* = 0.011, *q* = 0.044; # *p* = 0.041; bold values denote statistical significance at the *p* < 0.05.

**Table 4:** Comparison of the urinary concentrations of the analyzed EDCs among different sex.

	μg/L			μg/gCr		
	Male	Female	*p*	Male	Female	*p*
BPA	0.120 ± 0.042	0.248 ± 0.172	**0.039**	0.173 ± 0.154	1.132 ± 1.534	0.063
BPS	0.356 ± 0.420	0.311 ± 0.337	0.695	0.398 ± 0.400	1.060 ± 1.786	**0.043**
TCS	0.173 ± 0.176	0.148 ± 0.094	0.508	0.483 ± 1.326	0.591 ± 0.721	0.712
RCO	1.407 ± 1.848	1.943 ± 1.194	0.493	1.912 ± 2.451	4.256 ± 3.148	0.070

Bold values denote statistical significance at the *p* < 0.05.

## Data Availability

The datasets used and/or analyzed during the current study are available from the corresponding author on reasonable request.

## Supplementary Materials

The following supporting information can be downloaded at: https://www.mdpi.com/article/10.3390/medicina62071409/s1, Table S1: MS conditions for the GC-MS analysis of derivatized phenols. Table S2: Comparison of studied parameters between patients with urinary BPA levels above and below the LOQ in the total cohort and stratified by sex. Table S3: BMI- and age-adjusted *p*-values and Benjamini-Hochberg FDR-adjusted *q*-values for the regression analyses between urinary BPA concentrations (μg/L and μg/gCr) and the observed parameters. Table S4: Comparison of analyzed parameters between patients with urinary BPS levels above and below the LOQ in the total cohort and stratified by sex. Table S5: BMI- and age-adjusted *p*-values and Benjamini-Hochberg FDR-adjusted *q*-values for the regression analyses between urinary BPS concentrations (μg/L and μg/gCr) and the observed parameters. Table S6: Comparison of observed parameters between patients with urinary TCS levels above and below the LOQ in the total cohort and stratified by sex. Table S7: BMI- and age-adjusted *p*-values and Benjamini-Hochberg FDR-adjusted *q*-values for the regression analyses between urinary TCS concentrations (μg/L and μg/gCr) and the observed parameters. Table S8: Comparison of analyzed parameters between patients with urinary RCO levels above and below the LOQ in the total cohort and stratified by sex. Table S9: BMI- and age-adjusted *p*-values and Benjamini-Hochberg FDR-adjusted *q*-values for the regression analyses between urinary RCO concentrations (μg/L and μg/gCr) and the observed parameters. Figure S1: Box plot of urinary concentrations of the selected EDCs in (a) μg/L and (b) μg/gCr.
