# Supplementary material for: Phenolic Endocrine-Disrupting Chemical Exposure and Systemic Biomarker Variability in Patients with Lung Cancer
Source: Medicina (Kaunas). 2026 Jul 21;62(7):1409. doi: 10.3390/medicina62071409 (PMC13414361; doi:10.3390/medicina62071409)
Supplement: Supplementary file 1 [file medicina-62-01409-s001.zip › medicina-4412509-supplementary.pdf]

# Phenolic Endocrine-Disrupting Chemical Exposure and Systemic Biomarker Variability in Patients with Lung Cancer

**Larisa Đurić [1], Nataša Milošević [1], Maja Milanović [1,*], Danica Sazdanić-Velikić [2], Jana Pavlović [3], Milorad Španović [4] and Nataša Milić [1]**

[1] Faculty of Medicine, Department of Pharmacy, University of Novi Sad, 21000 Novi Sad, Serbia;
larisa.djuric@mf.uns.ac.rs (L.Đ.); natasa.milosevic@mf.uns.ac.rs (N.M.); natasa.milic@mf.uns.ac.rs (N.M.)
[2] Faculty of Medicine, Institute for Pulmonary Diseases of Vojvodina, Clinic for Pulmonary Oncology,
University of Novi Sad, 21204 Sremska Kamenica, Serbia; danica.sazdanic-velikic@mf.uns.ac.rs
[3] Faculty of Medicine Foča, University of East Sarajevo, 73300 Foča, Bosnia and Herzegovina; jana.stojanovic@ues.rs.ba
[4] Faculty of Medicine, Institute of Public Health of Vojvodina, University of Novi Sad, 21000 Novi Sad, Serbia; milorad.spanovic@mf.uns.ac.rs
* Correspondence: maja.milanovic@mf.uns.ac.rs

**Table S1.** MS conditions for the GC-MS analysis of derivatized phenols

| Analyte | Monitored ions (m/z) | | Retention time (min) | LOQ (μg/L) |
|---|---|---|---|---|
| | Quantifier | Qualifier | | |
| **BPA** | 357 | 372. 358 | 19.069 | 0.08 |
| **BPS** | 394 | 379. 182 | 22.910 | 0.10 |
| **TCS** | 345 | 360. 347 | 18.459 | 0.10 |
| **RCO** | 239 | 254. 73 | 11.217 | 0.10 |

**Table S2.** Comparison of studied parameters between patients with urinary BPA levels above and below the LOQ in the total cohort and stratified by sex.

| | **Total** | | | | **Male** | | | | **Female** | | | |
|---|---|---|---|---|---|---|---|---|---|---|---|---|
| Parameter | BPA ≥LOQ N=15 | BPA <LOQ N=175 | *p*-value | *q*-value* | BPA ≥LOQ N=10 | BPA <LOQ N=95 | *p*-value | *q*-value* | BPA ≥LOQ N=5* | BPA <LOQ N=80 | *p*-value | *q*-value* |
| Age | 65.867±8.618 | 65.651±8.541 | 0.926 | 1.000 | 63.600±7.619 | 67.421±8.417 | 0.172 | 0.641 | 70.400±9.529 | 63.550±8.254 | 0.078 | 0.320 |
| BMI | 25.268±4.162 | 25.846±4.877 | 0.746 | 1.000 | 24.957±3.762 | 25.018±4.430 | 0.961 | 0.980 | 25.909±5.293 | 24.641±5.383 | 0.611 | 0.892 |
| WC | 94.534±8.888 | 90.994±18.866 | 0.366 | 1.000 | 94.500±9.981 | 95.011±14.053 | 0.911 | 1.000 | **94.600±7.232** | **86.299±14.491** | **0.060** | 0.273 |
| Hip C | 99.733±9.550 | 98.162±10.964 | 0.592 | 1.000 | 95.700±7.804 | 97.556±9.737 | 0.562 | 1.000 | **107.800±7.759** | **98.870±12.271** | **0.059** | 0.273 |
| WHR | 0.952±0.082 | 0.926±0.102 | 0.348 | 1.000 | 0.987±0.062 | 0.972±0.084 | 0.575 | 1.000 | 0.880±0.072 | 0.872±0.094 | 0.866 | 0.900 |
| WtHR | 0.549±0.057 | 0.521±0.130 | 0.415 | 1.000 | 0.539±0.064 | 0.523±0.136 | 0.721 | 0.985 | **0.570±0.031** | **0.520±0.123** | **0.021** | 0.172 |
| BP systolic | 127.000±23.664 | 124.905±17.778 | 0.671 | 1.000 | 134.00±25.47 | 125.50±17.22 | 0.162 | 0.681 | 113.00±9.28 | 124.19±18.51 | 0.187 | 0.590 |
| BP diastolic | **77.067±15.040** | **72.299±9.964** | **0.092** | 1.000 | **77.60±17.76** | **71.80±9.28** | **0.096** | 0.492 | 76.00±8.94 | 72.88±10.74 | 0.528 | 0.891 |
| Pulse | 81.933±11.215 | 80.959±15.009 | 0.807 | 1.000 | 79.700±11.314 | 80.065±14.402 | 0.938 | 1.000 | 86.400±10.714 | 82.026±15.732 | 0.543 | 0.891 |
| RBC ($\times10^{12}$/L) | 4.665±0.461 | 4.453±0.586 | 0.175 | 1.000 | 4.613±0.474 | 4.522±0.676 | 0.679 | 1.000 | **4.767±0.465** | **4.370±0.443** | **0.056** | 0.302 |
| WBC ($\times10^{9}$/L) | 11.020±7.21 | 9.904±6.579 | 0.532 | 1.000 | 8.970±1.689 | 9.735±3.205 | 0.460 | 1.000 | 15.120±11.993 | 10.109±9.169 | 0.248 | 0.700 |
| Neu ($\times10^{9}$/L) | 8.337±7.096 | 6.814±2.718 | 0.439 | 1.000 | 6.417±1.723 | 7.103±2.871 | 0.483 | 1.000 | **11.794±11.597** | **6.457±2.488** | **0.002** | 0.027 |
| Neu (%) | 71.214±8.833 | 70.718±8.878 | 0.841 | 1.000 | 71.111±8.343 | 71.563±8.416 | 0.878 | 1.000 | 71.400±10.691 | 69.675±9.367 | 0.693 | 0.836 |
| Lym ($\times10^{9}$/L) | 1.791±0.544 | 1.735±0.724 | 0.781 | 1.000 | 1.541±0.394 | 1.624±0.645 | 0.707 | 1.000 | 2.240±0.511 | 1.873±0.793 | 0.312 | 0.711 |
| Lym (%) | 19.000±7.555 | 19.142±7.868 | 0.948 | 0.977 | 18.222±7.049 | 17.446±7.177 | 0.757 | 1.000 | 20.400±9.072 | 21.234±8.219 | 0.827 | 0.942 |
| Mon ($\times10^{9}$/L) | 0.785±0.428 | 0.735±0.291 | 0.551 | 1.000 | **0.698±0.164** | **0.807±0.279** | **0.096** | 0.492 | **0.942±0.703** | **0.644±0.281** | **0.044** | 0.301 |
| Mon (%) | 7.429±1.742 | 7.862±2.407 | 0.511 | 1.000 | 8.000±1.803 | 8.456±2.337 | 0.571 | 1.000 | 6.400±1.140 | 7.130±2.302 | 0.486 | 0.866 |
| Eos ($\times10^{9}$/L) | 0.154±0.194 | 0.152±0.231 | 0.984 | 0.984 | 0.196±0.231 | 0.177±0.294 | 0.855 | 1.000 | 0.078±0.064 | 0.122±0.108 | 0.376 | 0.811 |
| Eos (%) | 2.650±1.984 | 1.578±1.967 | 0.894 | 1.000 | 2.100±2.324 | 1.731±2.427 | 0.663 | 1.000 | 0.840±0.844 | 1.387±1.164 | 0.306 | 0.711 |
| Bas ($\times10^{9}$/L) | 0.050±0.034 | 0.060±0.035 | 0.302 | 1.000 | **0.041±0.019** | **0.062±0.036** | **0.011** | 0.226 | 0.066±0.051 | 0.057±0.034 | 0.580 | 0.881 |
| Bas (%) | 0.571±0.377 | 0.635±0.315 | 0.475 | 1.000 | 0.500±0.235 | 0.634±0.307 | 0.206 | 0.650 | 0.700±0.566 | 0.634±0.327 | 0.689 | 0.836 |
| Hgb | 135.733±10.721 | 129.578±16.675 | 0.162 | 1.000 | 135.40±10.83 | 130.57±17.70 | 0.400 | 1.000 | 136.40±11.72 | 128.37±15.36 | 0.256 | 0.700 |
| Hct | 0.401±0.039 | 0.387±0.046 | 0.257 | 1.000 | 0.394±0.041 | 0.391±0.049 | 0.828 | 1.000 | 0.413±0.035 | 0.386±0.042 | 0.107 | 0.366 |
| MCV | 86.133±4.274 | 86.878±8.966 | 0.751 | 1.000 | 85.700±4.968 | 86.398±11.019 | 0.844 | 1.000 | 87.000±2.646 | 87.461±5.540 | 0.854 | 0.932 |
| MCH | 29.227±2.031 | 29.291±2.500 | 0.923 | 1.000 | 29.520±2.293 | 29.186±2.675 | 0.705 | 1.000 | 028.640±1.398 | 29.419±2.280 | 0.454 | 0.878 |
| MCHC | 339.333±14.331 | 335.012±9.426 | 0.106 | 1.000 | **344.20±14.91** | **333.94±9.82** | **0.004** | 0.164 | **329.60±6.426** | **336.32±8.810** | **0.098** | 0.365 |
| PLT ($\times10^{9}$/L) | 292.267±84.884 | 324.427±116.414 | 0.297 | 1.000 | 287.00±71.02 | 317.48±116.05 | 0.418 | 1.000 | 302.80±116.88 | 332.88±117.05 | 0.579 | 0.881 |
| ALT | 0.373±0.202 | 0.366±0.382 | 0.938 | 0.997 | 0.339±0.164 | 0.335±0.246 | 0.956 | 0.985 | 0.442±0.270 | 0.403±0.270 | 0.864 | 0.910 |
| AST | 0.353±0.207 | 0.359±0.217 | 0.909 | 1.000 | 0.386±0.247 | 0.344±0.194 | 0.530 | 1.000 | 0.286±0.060 | 0.378±0.242 | 0.402 | 0.824 |
| GGT | 0.661±0.359 | 0.906±2.035 | 0.644 | 1.000 | 0.652±0.375 | 0.859±1.191 | 0.587 | 1.000 | 0.680±0.366 | 0.962±2.744 | 0.820 | 0.942 |
| LDH | 7.843±5.721 | 7.753±5.386 | 0.953 | 0.977 | 8.792±6.447 | 7.611±4.063 | 0.416 | 1.000 | 5.470±2.566 | 7.933±6.728 | 0.471 | 0.866 |
| ALP | 1.328±0.471 | 1.628±0.894 | 0.202 | 1.000 | **1.206±0.486** | **1.611±0.679** | **0.071** | 0.485 | 1.572±0.366 | 1.649±1.101 | 0.878 | 0.900 |
| Total bilirubin | 7.713±2.443 | 7.245±3.608 | 0.623 | 1.000 | 8.080±2.456 | 7.939±4.058 | 0.915 | 1.000 | 6.980±2.490 | 6.409±2.783 | 0.656 | 0.862 |
| Direct bilirubin | 2.650±0.733 | 2.544±1.044 | 0.709 | 1.000 | 2.720±0.781 | 2.730±1.185 | 0.980 | 0.980 | 2.475±0.665 | 2.307±0.777 | 0.673 | 0.856 |
| Urea | 6.853±5.063 | 6.041±2.372 | 0.259 | 1.000 | 5.590±1.187 | 6.618±2.246 | 0.166 | 0.641 | **9.380±8.361** | **5.347±2.347** | **0.004** | 0.041 |

| | | | | | | | | | | | | |
|---|---|---|---|---|---|---|---|---|---|---|---|---|
| Serum creatinine | 77.533±25.593 | 71.820±19.872 | 0.298 | 1.000 | **71.10±9.219** | **79.365±19.357** | **0.030** | 0.262 | **90.400±42.318** | **62.747±16.479** | **0.002** | 0.027 |
| CRP | 27.820±32.234 | 36.289±48.910 | 0.512 | 1.000 | **20.790±27.546** | **46.546±56.057** | **0.024** | 0.308 | 41.880±39.480 | 24.191±35.536 | 0.287 | 0.735 |
| CK | 1.323±0.764 | 1.215±0.952 | 0.668 | 1.000 | 1.532±0.796 | 1.320±1.050 | 0.536 | 1.000 | 0.906±0.538 | 1.081±0.797 | 0.631 | 0.869 |
| CK-MB | 0.544±0.432 | 0.381±0.504 | 0.243 | 1.000 | 0.601±0.494 | 0.371±0.543 | 0.203 | 0.650 | 0.400±0.192 | 0.394±0.451 | 0.978 | 0.978 |
| Uric acid | 312.357±130.624 | 295.206±95.452 | 0.527 | 1.000 | **253.89±93.45** | **322.01±89.27** | **0.032** | 0.262 | **417.60±128.26** | **262.56±90.74** | **0.001** | 0.041 |
| Glucose | 7.127±2.260 | 6.676±2.644 | 0.523 | 1.000 | 7.270±2.334 | 6.915±3.044 | 0.721 | 0.985 | 6.840±2.337 | 6.389±2.047 | 0.636 | 0.868 |

* q-values were obtained using the Benjamini–Hochberg false discovery rate (FDR) correction for multiple comparisons

**Table S3.** BMI- and age-adjusted *p*-values and Benjamini-Hochberg FDR-adjusted *q*-values for the regression analyses between urinary BPA concentrations (µg/L and µg/gCr) and the observed parameters.

| Parameter | BPA (µg/L) | | | | | | BPA(µg/gCr) | | | | | |
|---|---|---|---|---|---|---|---|---|---|---|---|---|
| | Total N=15 | | Male N=10 | | Female N=5 | | Total N=15 | | Male N=10 | | Female N=5 | |
| | *p*-value | *q*-value | *p*-value | *q*-value | *p*-value | *q*-value | *p*-value | *q*-value | *p*-value | *q*-value | *p*-value | *q*-value |
| WC | 0.778 | 0.954 | 0.934 | 0.982 | 0.117 | 1.000 | 0.640 | 0.924 | 0.983 | 0.983 | 0.173 | 1.000 |
| Hip C | 0.295 | 0.894 | 0.903 | 0.978 | 0.712 | 0.915 | 0.393 | 1.000 | 0.903 | 1.000 | 0.656 | 0.919 |
| WHR | 0.142 | 0.692 | 0.722 | 0.917 | 0.714 | 0.887 | 0.161 | 1.000 | 0.737 | 1.000 | 0.770 | 0.970 |
| WtHR | 0.335 | 0.787 | 0.286 | 1.000 | 0.145 | 1.000 | 0.532 | 0.988 | 0.733 | 1.000 | 0.202 | 1.000 |
| BP systolic | 0.317 | 0.817 | 0.553 | 1.000 | 0.714 | 0.871 | 0.773 | 0.968 | 0.177 | 0.725 | 0.771 | 0.948 |
| BP diastolic | 0.591 | 0.958 | 0.710 | 0.990 | 0.157 | 0.937 | 0.254 | 0.991 | **0.042** | 0.741 | 0.213 | 1.000 |
| Pulse | 0.307 | 0.824 | 0.124 | 1.000 | 0.160 | 0.937 | 0.430 | 1.000 | 0.388 | 1.000 | 0.216 | 0.936 |
| RBC ($\times10^{12}$/L) | 0.537 | 0.911 | 0.673 | 1.000 | 0.341 | 0.777 | 0.210 | 1.000 | 0.365 | 1.000 | 0.285 | 0.729 |
| WBC ($\times10^{9}$/L) | 0.361 | 0.782 | 0.546 | 1.000 | 0.266 | 0.923 | 0.922 | 0.942 | 0.767 | 0.995 | 0.210 | 1.000 |
| Neu ($\times10^{9}$/L) | 0.453 | 0.841 | 0.243 | 1.000 | 0.317 | 0.817 | 0.942 | 0.942 | 0.495 | 0.899 | 0.261 | 0.855 |
| Neu (%) | 0.783 | 0.934 | 0.236 | 1.000 | 0.331 | 0.777 | 0.483 | 1.000 | 0.137 | 0.886 | 0.275 | 0.766 |
| Lym ($\times10^{9}$/L) | 0.251 | 0.922 | 0.729 | 0.917 | 0.920 | 1.000 | 0.378 | 1.000 | 0.322 | 1.000 | 0.976 | 0.994 |
| Lym (%) | 0.404 | 0.829 | 0.359 | 1.000 | 0.464 | 0.769 | 0.239 | 0.991 | 0.174 | 0.767 | 0.408 | 0.758 |
| Mon ($\times10^{9}$/L) | 0.861 | 0.933 | 0.685 | 1.000 | 0.319 | 0.798 | 0.484 | 0.996 | 0.760 | 0.997 | 0.263 | 0.825 |
| Mon (%) | 0.138 | 0.692 | 0.500 | 1.000 | 0.846 | 0.991 | 0.211 | 1.000 | 0.432 | 0.936 | 0.790 | 0.934 |
| Eos ($\times10^{9}$/L) | 0.618 | 0.927 | 0.680 | 1.000 | **0.040** | 0.984 | 0.750 | 0.972 | 0.507 | 0.865 | **0.096** | 1.000 |
| Eos (%) | 0.704 | 0.947 | 0.574 | 1.000 | **0.049** | 0.670 | 0.884 | 0.967 | 0.485 | 0.919 | 0.105 | 1.000 |
| Bas ($\times10^{9}$/L) | **0.032** | 0.494 | 0.547 | 1.000 | 0.228 | 0.996 | 0.008 | 0.312 | 0.186 | 0.725 | 0.902 | 0.971 |
| Bas (%) | **0.086** | 0.686 | 0.711 | 0.964 | 0.313 | 0.866 | 0.029 | 0.566 | 0.943 | 1.000 | 0.369 | 0.757 |
| Hgb | 0.343 | 0.782 | 0.286 | 1.000 | 0.496 | 0.782 | 0.322 | 1.000 | 0.672 | 1.000 | 0.440 | 0.746 |
| Hct | 0.815 | 0.935 | 0.347 | 1.000 | 0.449 | 0.825 | 0.596 | 1.000 | 0.429 | 0.936 | 0.393 | 0.758 |
| MCV | 0.437 | 0.841 | 0.294 | 1.000 | 0.375 | 0.769 | 0.146 | 1.000 | 0.954 | 0.995 | 0.431 | 0.746 |
| MCH | 0.790 | 0.934 | 0.590 | 1.000 | 0.469 | 0.769 | 0.588 | 1.000 | 0.484 | 0.946 | 0.526 | 0.821 |
| MCHC | 0.130 | 0.769 | 0.963 | 0.963 | 0.604 | 0.917 | 0.461 | 1.000 | 0.134 | 0.891 | 0.660 | 0.919 |
| PLT ($\times10^{9}$/L) | **0.038** | 0.494 | **0.009** | 0.176 | 0.463 | 0.793 | 0.212 | 1.000 | 0.357 | 1.000 | 0.519 | 0.821 |
| ALT | 0.643 | 0.898 | 0.717 | 0.939 | 0.219 | 1.000 | 0.514 | 0.988 | 0.418 | 0.984 | 0.275 | 0.741 |
| AST | 0.859 | 0.933 | 0.630 | 1.000 | 0.303 | 0.917 | 0.896 | 0.944 | 0.875 | 1.000 | 0.247 | 0.925 |
| GGT | 0.905 | 0.954 | 0.767 | 0.935 | 0.270 | 0.923 | 0.676 | 0.933 | 0.405 | 1.000 | 0.213 | 0.936 |
| LDH | 0.298 | 0.855 | 0.516 | 1.000 | 0.899 | 1.000 | 0.794 | 0.957 | 0.671 | 1.000 | 0.874 | 0.974 |
| ALP | **0.021** | 0.624 | 0.291 | 1.000 | 0.243 | 0.991 | 0.077 | 1.000 | 0.159 | 0.848 | 0.299 | 0.709 |
| Total bilirubin | 0.987 | 0.988 | 0.890 | 0.978 | 0.699 | 0.942 | 0.810 | 0.943 | 0.585 | 0.951 | 0.755 | 1.000 |
| Direct bilirubin | 0.772 | 0.979 | 0.850 | 1.000 | 0.973 | 1.000 | 0.617 | 0.966 | 0.791 | 0.995 | 0.817 | 0.937 |
| Urea | 0.645 | 0.898 | 0.875 | 0.992 | **0.048** | 0.670 | 0.619 | 0.960 | **0.078** | 0.761 | **0.008** | 0.312 |
| Serum creatinine | 0.260 | 0.922 | 0.220 | 1.000 | 0.365 | 0.769 | 0.694 | 0.933 | **0.024** | 0.819 | 0.309 | 0.709 |
| CRP | 0.249 | 0.979 | **0.009** | 0.176 | 0.411 | 0.802 | 0.893 | 0.944 | 0.925 | 1.000 | 0.355 | 0.757 |
| CK | 0.988 | 0.988 | 0.957 | 0.963 | 0.950 | 1.000 | 0.640 | 0.924 | 0.510 | 0.865 | 0.994 | 0.994 |
| CK-MB | 0.614 | 0.927 | 0.672 | 1.000 | 0.698 | 0.955 | 0.822 | 0.943 | 0.952 | 1.000 | 0.921 | 0.971 |
| Uric acid | **0.088** | 0.686 | 0.689 | 1.000 | 0.722 | 0.871 | 0.296 | 1.000 | **0.057** | 0.741 | 0.778 | 0.934 |
| Glucose | 0.511 | 0.906 | 0.239 | 1.000 | 0.630 | 0.923 | 0.485 | 0.996 | 0.969 | 0.983 | 0.574 | 0.861 |

**Table S4.** Comparison of analyzed parameters between patients with urinary BPS levels above and below the LOQ in the total cohort and stratified by sex.

| | **Total** | | | | **Male** | | | | **Female** | | | |
|---|---|---|---|---|---|---|---|---|---|---|---|---|
| Parameter | BPS ≥LOQ N=52 | BPS <LOQ N=138 | *p*-value | *q*-value* | BPS ≥LOQ N=34 | BPS <LOQ N=71 | *p*-value | *q*-value* | BPS ≥LOQ N=18 | BPS <LOQ N=67 | *p*-value | *q*-value* |
| Age | 67.135±9.140 | 65.116±8.247 | 0.146 | 0.665 | 67.853±9.056 | 66.676±8.085 | 0.504 | 0.898 | 65.778±9.403 | 63.463±8.153 | 0.304 | 0.834 |
| BMI | 24.740±4.531 | 24.933±4.935 | 0.807 | 0.945 | 25.656±4.585 | 24.698±4.235 | 0.295 | 0.930 | 23.012±3.993 | 25.181±5.605 | 0.128 | 0.875 |
| WC | 91.412±15.004 | 91.237±14.333 | 0.942 | 0.950 | 95.618±14.871 | 94.621±13.101 | 0.732 | 0.857 | 83.000±11.603 | 87.800±14.805 | 0.219 | 0.752 |
| Hip C | 97.843±10.778 | 98.466±10.899 | 0.729 | 0.967 | 97.059±11.073 | 97.530±8.741 | 0.816 | 0.880 | 99.412±10.308 | 99.415±12.723 | 0.999 | 0.999 |
| WHR | 0.935±0.112 | 0.925±0.096 | 0.575 | 0.907 | 0.984±0.089 | 0.968±0.078 | 0.346 | 0.962 | **0.836±0.084** | **0.882±0.093** | **0.064** | 1.000 |
| WtHR | 0.529±0.111 | 0.522±0.131 | 0.743 | 0.943 | **0.553±0.087** | **0.510±0.146** | **0.065** | 0.666 | 0.482±0.137 | 0.534±0.114 | 0.104 | 1.000 |
| BP systolic | 124.137±17.899 | 125.436±18.447 | 0.667 | 1.000 | 124.42±19.50 | 127.25±17.63 | 0.467 | 0.870 | 123.61±15.03 | 123.48±19.24 | 0.979 | 0.999 |
| BP diastolic | 72.220±10.316 | 72.871±10.603 | 0.710 | 0.996 | 71.44±9.54 | 72.82±10.89 | 0.539 | 0.921 | 73.61±11.73 | 72.92±10.38 | 0.810 | 0.949 |
| Pulse | 79.431±11.323 | 81.654±15.818 | 0.360 | 0.777 | 78.333±11.327 | 80.841±15.235 | 0.403 | 0.865 | 81.444±11.356 | 82.531±16.499 | 0.794 | 0.957 |
| RBC ($\times10^{12}$/L) | **4.640±0.762** | **4.407±0.482** | **0.014** | 0.390 | **4.716±0.885** | **4.441±0.499** | **0.045** | 0.615 | 4.488±0.404 | 4.367±0.463 | 0.339 | 0.774 |
| WBC ($\times10^{9}$/L) | 9.866±4.605 | 10.040±7.239 | 0.873 | 0.967 | 9.493±2.678 | 9.743±3.291 | 0.701 | 0.871 | 10.612±7.114 | 10.360±9.889 | 0.922 | 1.000 |
| Neu ($\times10^{9}$/L) | 7.084±4.397 | 6.871±2.720 | 0.693 | 1.000 | 6.838±2.560 | 7.139±2.905 | 0.611 | 0.992 | 7.561±6.763 | 70.600±8.981 | 0.340 | 0.744 |
| Neu (%) | **68.760±9.328** | **71.489±8.590** | **0.062** | 0.492 | 69.849±8.624 | 72.303±8.195 | 0.165 | 0.964 | 66.657±10.511 | 70.600±8.981 | 0.123 | 0.875 |
| Lym ($\times10^{9}$/L) | 1.836±0.626 | 1.704±0.738 | 0.265 | 0.700 | 1.698±0.590 | 1.579±0.643 | 0.371 | 0.902 | 2.103±0.622 | 1.841±0.814 | 0.220 | 0.752 |
| Lym (%) | 20.300±7.843 | 18.701±7.803 | 0.218 | 0.638 | 18.636±7.110 | 16.991±7.137 | 0.276 | 0.930 | 23.529±8.397 | 20.569±8.120 | 0.188 | 0.938 |
| Mon ($\times10^{9}$/L) | **0.799±0.316** | **0.716±0.295** | **0.074** | 0.433 | 0.799±0.187 | 0.799±0.304 | 0.989 | 0.989 | **7.882±2.497** | **6.877±2.154** | **0.043** | 1.000 |
| Mon (%) | **8.340±2.300** | **7.642±2.366** | **0.072** | 0.433 | 8.576±2.194 | 8.342±2.348 | 0.631 | 0.958 | 7.882±2.497 | 6.877±2.154 | 0.101 | 1.000 |
| Eos ($\times10^{9}$/L) | 0.173±0.191 | 0.145±0.241 | 0.456 | 0.885 | 0.211±0.221 | 0.163±0.315 | 0.435 | 0.849 | 0.099±0.069 | 0.124±0.114 | 0.382 | 0.750 |
| Eos (%) | 1.892±2.070 | 1.469±1.917 | 0.194 | 0.663 | 2.202±2.381 | 1.559±2.412 | 0.207 | 0.849 | 1.288±1.096 | 1.371±1.142 | 0.794 | 0.949 |
| Bas ($\times10^{9}$/L) | 0.580±0.035 | 0.060±0.349 | 0.759 | 0.943 | 0.059±0.036 | 0.062±0.035 | 0.667 | 0.869 | 0.057±0.035 | 0.058±0.035 | 0.947 | 1.000 |
| Bas (%) | 0.627±0.344 | 0.632±0.312 | 0.928 | 0.966 | 0.604±0.317 | 0.631±0.298 | 0.678 | 0.869 | 0.671±0.396 | 0.632±0.328 | 0.683 | 1.000 |
| Hgb | 132.216±14.935 | 129.270±16.822 | 0.273 | 0.700 | 132.12±16.35 | 130.51±17.66 | 0.655 | 0.895 | 132.41±12.06 | 127.94±15.91 | 0.283 | 0.890 |
| Hct | 0.396±0.042 | 0.385±0.047 | 0.127 | 0.651 | 0.397±0.045 | 0.388±0.049 | 0.422 | 0.849 | 0.395±0.035 | 0.381±0.044 | 0.206 | 0.861 |
| MCV | 87.177±6.514 | 86.685±9.377 | 0.731 | 0.952 | 86.618±7.402 | 86.195±11.849 | 0.849 | 0.893 | 88.294±4.195 | 87.212±5.677 | 0.465 | 0.867 |
| MCH | 29.106±2.650 | 29.353±2.394 | 0.542 | 0.918 | 28.871±2.968 | 29.384±2.462 | 0.352 | 0.951 | 29.576±1.848 | 29.320±2.338 | 0.676 | 1.000 |
| MCHC | 333.726±9.894 | 335.941±9.902 | 0.170 | 0.697 | 332.91±10.42 | 335.87±10.84 | 0.188 | 0.861 | 335.35±8.80 | 336.06±8.865 | 0.770 | 0.999 |
| PLT ($\times10^{9}$/L) | 308.941±104.883 | 326.672±117.712 | 0.346 | 0.777 | 308.91±107.20 | 317.29±115.81 | 0.723 | 0.857 | 309.00±103.31 | 336.76±119.79 | 0.384 | 0.750 |
| ALT | 0.355±0.292 | 0.371±0.398 | 0.794 | 0.945 | 0.379±0.335 | 0.313±0.171 | 0.189 | 0.849 | 0.309±0.187 | 0.672±2.004 | 0.345 | 0.744 |
| AST | 0.364±0.187 | 0.357±0.226 | 0.842 | 0.959 | **0.395±0.221** | **0.326±0.184** | **0.096** | 0.656 | 0.306±0.073 | 0.432±0.541 | 0.175 | 0.964 |
| GGT | 0.721±0.848 | 0.951±2.244 | 0.475 | 0.885 | 0.791±0.895 | 0.863±1.247 | 0.764 | 0.862 | 0.589±0.759 | 1.045±2.987 | 0.524 | 0.934 |
| LDH | 7.803±3.878 | 7.745±5.882 | 0.950 | 0.950 | 7.997±4.028 | 7.598±4.494 | 0.655 | 0.882 | 7.373±3.623 | 7.909±7.159 | 0.780 | 0.986 |
| ALP | 1.543±0.835 | 1.627±0.885 | 0.560 | 0.907 | 1.523±0.791 | 1.593±0.610 | 0.629 | 0.958 | 1.578±0.932 | 1.663±1.113 | 0.768 | 1.000 |
| Total bilirubin | 7.800±4.171 | 7.085±3.245 | 0.214 | 0.638 | 8.426±4.656 | 7.725±3.537 | 0.394 | 0.870 | 6.617±2.803 | 6.396±2.763 | 0.765 | 1.000 |
| Direct bilirubin | 2.566±0.905 | 2.546±1.067 | 0.908 | 0.976 | 2.712±0.964 | 2.737±1.236 | 0.917 | 0.940 | 2.256±0.691 | 2.331±0.792 | 0.732 | 1.000 |
| Urea | 6.519±3.301 | 5.948±2.385 | 0.190 | 0.663 | 6.662±2.380 | 6.452±2.162 | 0.654 | 0.926 | 6.250±4.645 | 5.406±2.508 | 0.305 | 0.815 |

| | | | | | | | | | | | | |
|---|---|---|---|---|---|---|---|---|---|---|---|---|
| Serum creatinine | **77.500±22.899** | **70.290±19.027** | **0.029** | 0.308 | 81.706±20.008 | 77.080±18.088 | 0.239 | 0.891 | 69.556±26.340 | 62.985±17.336 | 0.210 | 0.816 |
| CRP | 30.159±38.620 | 37.674±50.795 | 0.340 | 0.788 | **32.639±38.882** | **49.464±59.911** | **0.092** | 0.656 | 25.611±38.823 | 25.159±35.204 | 0.962 | 1.000 |
| CK | **1.462±1.199** | **1.128±0.792** | **0.030** | 0.308 | **1.717±1.370** | **1.155±0.751** | **0.008** | 0.328 | 0.979±0.538 | 1.097±0.843 | 0.578 | 0.954 |
| CK-MB | 0.441±0.500 | 0.376±0.500 | 0.447 | 0.890 | 0.416±0.424 | 0.382±0.592 | 0.778 | 0.862 | 0.495±0.651 | 0.369±0.366 | 0.318 | 0.815 |
| Uric acid | **323.725±100.012** | **286.277±94.593** | **0.019** | 0.390 | **345.70±81.39** | **302.26±92.79** | **0.023** | 0.472 | 283.44±119.55 | 268.71±94.10 | 0.582 | 0.954 |
| Glucose | 6.512±1.951 | 6.788±2.827 | 0.518 | 0.923 | 6.574±2.011 | 7.128±3.339 | 0.374 | 0.897 | 6.394±1.884 | 6.421±2.110 | 0.961 | 1.000 |

q-values were obtained using the Benjamini–Hochberg FDR correction for multiple comparisons

**Table S5.** BMI- and age-adjusted *p*-values and Benjamini-Hochberg FDR-adjusted *q*-values for the regression analyses between urinary BPS concentrations (µg/L and µg/gCr) and the observed parameters.

| Parameter | BPS (µg/L) | | | | | | BPS (µg/gCr) | | | | | |
|---|---|---|---|---|---|---|---|---|---|---|---|---|
| | Total N=52 | | Male N=34 | | Female N=17 | | Total N=52 | | Male N=34 | | Female N=17 | |
| | *p*-value | *q*-value | *p*-value | *q*-value | *p*-value | *q*-value | *p*-value | *q*-value | *p*-value | *q*-value | *p*-value | *q*-value |
| WC | **0.056** | 0.902 | 0.206 | 0.579 | 0.127 | 0.991 | 0.896 | 0.991 | 0.650 | 1.000 | 0.589 | 1.000 |
| Hip C | 0.575 | 0.869 | 0.388 | 0.759 | 0.806 | 1.000 | 0.163 | 0.706 | 0.344 | 1.000 | 0.809 | 1.000 |
| WHR | 0.237 | 0.822 | 0.732 | 0.878 | 0.099 | 1.000 | 0.262 | 1.000 | 0.472 | 0.938 | 0.752 | 1.000 |
| WtHR | 0.245 | 0.937 | 0.446 | 0.725 | 0.285 | 1.000 | 0.613 | 1.000 | 0.939 | 0.990 | 0.687 | 1.000 |
| BP systolic | 0.658 | 0.945 | 0.743 | 0.878 | 0.826 | 0.976 | 0.823 | 1.000 | 0.886 | 0.960 | 0.584 | 1.000 |
| BP diastolic | 0.509 | 0.814 | 0.874 | 0.942 | 0.326 | 1.000 | **0.036** | 0.419 | 0.668 | 1.000 | **0.088** | 0.878 |
| Pulse | 0.301 | 1.000 | 0.408 | 0.723 | 0.572 | 1.000 | **0.073** | 0.475 | 0.438 | 1.000 | 0.186 | 1.000 |
| RBC ($\times10^{12}$/L) | 0.916 | 0.795 | 0.951 | 0.951 | 0.647 | 1.000 | 0.554 | 1.000 | 0.481 | 0.938 | 0.951 | 0.976 |
| WBC ($\times10^{9}$/L) | 0.313 | 0.786 | 0.168 | 0.617 | 0.507 | 1.000 | 0.338 | 1.000 | 0.062 | 1.000 | 0.694 | 1.000 |
| Neu ($\times10^{9}$/L) | 0.383 | 0.557 | 0.174 | 0.617 | 0.532 | 1.000 | 0.434 | 1.000 | 0.081 | 1.000 | 0.716 | 1.000 |
| Neu (%) | 0.100 | 0.984 | 0.102 | 0.568 | 0.255 | 1.000 | 0.751 | 1.000 | 0.203 | 1.000 | 0.349 | 1.000 |
| Lym ($\times10^{9}$/L) | 0.932 | 0.924 | 0.433 | 0.725 | 0.214 | 1.000 | 0.135 | 0.726 | 0.821 | 1.000 | 0.474 | 1.000 |
| Lym (%) | 0.220 | 0.939 | 0.138 | 0.672 | 0.530 | 1.000 | 0.732 | 1.000 | 0.262 | 1.000 | 0.519 | 1.000 |
| Mon ($\times10^{9}$/L) | 0.706 | 0.400 | 0.343 | 0.833 | 0.502 | 1.000 | 0.564 | 1.000 | 0.577 | 1.000 | 0.698 | 1.000 |
| Mon (%) | **0.041** | 0.939 | 0.696 | 0.902 | **0.003** | 0.059 | **0.022** | 0.429 | 0.330 | 1.000 | **0.090** | 0.858 |
| Eos ($\times10^{9}$/L) | 0.722 | 0.924 | 0.389 | 0.743 | 0.315 | 1.000 | 0.782 | 1.000 | 0.848 | 0.969 | 0.310 | 1.000 |
| Eos (%) | 0.545 | 0.995 | 0.385 | 0.796 | 0.880 | 0.983 | 0.658 | 1.000 | 0.828 | 1.000 | 0.871 | 0.967 |
| Bas ($\times10^{9}$/L) | 0.995 | 0.882 | **0.040** | 0.260 | **0.001** | 0.039 | **0.016** | 0.429 | 0.371 | 0.963 | **0.001** | 0.039 |
| Bas (%) | 0.588 | 0.804 | **0.022** | 0.172 | 0.108 | 0.991 | **0.052** | 0.406 | 0.215 | 1.000 | **0.069** | 1.000 |
| Hgb | 0.253 | 0.868 | 0.194 | 0.618 | 0.948 | 0.973 | 0.727 | 1.000 | 0.870 | 0.960 | 0.622 | 1.000 |
| Hct | 0.178 | 0.937 | 0.155 | 0.655 | 0.882 | 0.983 | 0.857 | 1.000 | 0.835 | 1.000 | 0.846 | 1.000 |
| MCV | 0.673 | 1.000 | 0.538 | 0.793 | 0.548 | 1.000 | 0.823 | 1.000 | 0.751 | 1.000 | 0.779 | 1.000 |
| MCH | 0.824 | 0.882 | 0.628 | 0.845 | 0.423 | 1.000 | 0.952 | 0.993 | 0.779 | 1.000 | 0.480 | 1.000 |
| MCHC | 0.578 | 0.804 | 0.922 | 0.946 | 0.376 | 1.000 | 0.498 | 1.000 | 0.971 | 0.977 | 0.240 | 1.000 |
| PLT ($\times10^{9}$/L) | 0.268 | 0.924 | 0.208 | 0.579 | 0.801 | 1.000 | 0.972 | 0.972 | 0.162 | 1.000 | 0.329 | 1.000 |
| ALT | 0.535 | 1.000 | 0.493 | 0.769 | 0.824 | 0.976 | 0.389 | 1.000 | 0.365 | 0.965 | 0.541 | 1.000 |
| AST | 0.863 | 0.156 | 0.815 | 0.935 | 0.917 | 0.993 | 0.388 | 1.000 | 0.469 | 0.969 | 0.423 | 1.000 |
| GGT | **0.004** | 0.400 | **0.001** | 0.020 | 0.709 | 0.988 | 0.861 | 1.000 | 0.312 | 1.000 | 0.742 | 1.000 |
| LDH | **0.040** | 0.759 | **0.007** | 0.068 | 0.760 | 1.000 | 0.706 | 1.000 | 0.395 | 0.963 | 0.852 | 0.996 |
| ALP | 0.326 | 0.817 | 0.236 | 0.614 | 0.694 | 1.000 | 0.915 | 0.991 | 0.846 | 0.973 | 0.868 | 0.971 |
| Total bilirubin | 0.419 | 0.984 | 0.573 | 0.798 | 0.408 | 1.000 | 0.490 | 1.000 | 0.264 | 1.000 | 0.893 | 0.967 |
| Direct bilirubin | 0.934 | 0.987 | 0.894 | 0.942 | 0.639 | 1.000 | 0.470 | 1.000 | 0.716 | 1.000 | 0.749 | 1.000 |
| Urea | 0.962 | 1.000 | 0.717 | 0.892 | 0.610 | 1.000 | 0.968 | 0.972 | 0.810 | 1.000 | 0.918 | 0.968 |
| Serum creatinine | 0.882 | 1.000 | 0.400 | 0.723 | 0.235 | 1.000 | 0.703 | 1.000 | 0.557 | 1.000 | 0.282 | 1.000 |
| CRP | 0.837 | 0.758 | 0.864 | 0.947 | 0.696 | 0.988 | 0.893 | 0.998 | 0.977 | 0.977 | 0.988 | 0.988 |
| CK | 0.350 | 0.377 | 0.363 | 0.833 | 0.973 | 0.973 | 0.812 | 1.000 | 0.296 | 1.000 | 0.658 | 1.000 |
| CK-MB | **0.058** | 0.293 | **0.001** | 0.020 | 0.942 | 0.973 | 0.675 | 1.000 | 0.607 | 1.000 | 0.478 | 1.000 |
| Uric acid | **0.015** | 0.758 | **0.005** | 0.065 | 0.210 | 1.000 | **0.043** | 0.406 | **0.024** | 0.936 | 0.110 | 0.858 |
| Glucose | 0.331 | 0.377 | 0.549 | 0.793 | 0.654 | 1.000 | 0.149 | 0.706 | 0.246 | 1.000 | 0.418 | 1.000 |

**Table S6.** Comparison of observed parameters between patients with urinary TCS levels above and below the LOQ in the total cohort and stratified by sex.

| | **Total** | | | | **Male** | | | | **Female** | | | |
|---|---|---|---|---|---|---|---|---|---|---|---|---|
| Parameter | TCS ≥LOQ N=56 | TCS <LOQ N=134 | *p*-value | *q*-value* | TCS ≥LOQ N=29 | TCS <LOQ N=76 | *p*- value | *q*-value* | TCS ≥LOQ N=27 | TCS <LOQ N=58 | *p*-value | *q*-value* |
| Age | 66.321±9.094 | 65.396±8.294 | 0.496 | 0.813 | 69.345±6.662 | 66.184±8.840 | **0.052** | 0.355 | 63.074±10.295 | 64.362±7.459 | 0.515 | 1.000 |
| BMI | 25.460±4.749 | 24.633±4.840 | 0.283 | 0.725 | 25.423±3.823 | 24.852±4.556 | 0.551 | 0.763 | 25.499±5.654 | 24.345±5.216 | 0.359 | 1.000 |
| WC | **94.056±13.664** | **90.118±14.710** | **0.094** | 0.964 | 97.643±13.381 | 93.917±13.718 | 0.222 | 0.535 | 90.192±13.133 | 85.232±14.613 | 0.144 | 1.000 |
| Hip C | 99.815±10.574 | 97.649±10.926 | 0.219 | 0.931 | 97.714±9.645 | 97.236±9.569 | 0.823 | 0.877 | 102.08±11.29 | 98.18±12.53 | 0.180 | 1.000 |
| WHR | 0.942±0.090 | 0.9223±0.104 | 0.233 | 0.738 | **0.998±0.080** | **0.964±0.082** | **0.063** | 0.333 | 0.881±0.055 | 0.869±0.106 | 0.574 | 1.000 |
| WtHR | 0.538±0.131 | 0.518±0.124 | 0.308 | 0.743 | 0.540±0.126 | 0.519±0.133 | 0.462 | 0.702 | 0.536±0.138 | 0.516±0.112 | 0.479 | 1.000 |
| BP systolic | 125.982±19.747 | 124.70±17.669 | 0.666 | 0.827 | 129.82±20.88 | 125.01±17.06 | 0.236 | 0.509 | 121.84+±28.93 | 124.29±18.60 | 0.578 | 1.000 |
| BP diastolic | **74.793±10.869** | **71.830±10.278** | **0.084** | 0.964 | **75.259±12.38** | **71.315±9.51** | **0.094** | 0.312 | 74.31±9.19 | 72.50±11.25 | 0.477 | 1.000 |
| Pulse | 78.926±13.682 | 81.915±15.086 | 0.210 | 0.998 | **76.429±11.043** | **81.392±14.914** | **0.072** | 0.295 | 81.615±15.832 | 82.607±15.419 | 0.789 | 1.000 |
| RBC (×10$^{12}$/L) | 4.478±0.475 | 4.467±0.618 | 0.902 | 0.948 | 4.506±0.558 | 4.540±0.695 | 0.814 | 0.888 | 4.447±0.369 | 4.369±0.486 | 0.468 | 1.000 |
| WBC (×10$^{9}$/L) | 9.131±2.675 | 10.349±7.660 | 0.252 | 0.689 | **8.365±2.043** | **10.158±3.290** | **0.007** | 0.287 | 9.984±3.056 | 10.605±11.121 | 0.781 | 1.000 |
| Neu (×10$^{9}$/L) | 6.636±2.353 | 7.048±3.547 | 0.433 | 0.809 | **6.051±1.768** | **7.409±3.012** | **0.027** | 0.262 | 7.266±2.751 | 6.558±4.143 | 0.430 | 1.000 |
| Neu (%) | 71.982±7.793 | 70.254±9.231 | 0.228 | 0.796 | 72.321±6.475 | 71.230±8.989 | 0.558 | 0.741 | 71.615±9.122 | 68.929±9.469 | 0.230 | 1.000 |
| Lym (×10$^{9}$/L) | 1.641±0.699 | 1.780±0.714 | 0.227 | 0.850 | **1.384±0.504** | **1.702±0.647** | **0.021** | 0.277 | 1.918±0.780 | 1.885±0.789 | 0.863 | 0.931 |
| Lym (%) | 18.389±6.747 | 19.435±8.230 | 0.409 | 0.838 | 16.857±5.549 | 17.755±7.656 | 0.572 | 0.733 | 20.038±7.602 | 21.714±8.497 | 0.393 | 1.000 |
| Mon (×10$^{9}$/L) | **0.663±0.221** | **0.768±0.325** | **0.036** | 1.000 | **0.718±0.170** | **0.829±0.296** | **0.020** | 0.287 | 0.610±0.257 | 0.686±0.347 | 0.326 | 1.000 |
| Mon (%) | 7.574±2.492 | 7.934±2.309 | 0.347 | 0.790 | 8.857±2.172 | 8.254±2328 | 0.236 | 0.509 | **6.192±2.060** | **7.500±2.232** | **0.013** | 0.533 |
| Eos (×10$^{9}$/L) | 0.119±0.123 | 0.166±0.259 | 0.204 | 1.000 | **0.110±0.133** | **0.205±0.324** | **0.032** | 0.262 | 0.131±0.112 | 0.113±0.103 | 0.471 | 1.000 |
| Eos (%) | 1.314±1.272 | 1.692±2.178 | 0.234 | 0.697 | **1.224±1.354** | **1.952±2.677** | **0.068** | 0.295 | 1.411±1.197 | 1.327±1.139 | 0.758 | 1.000 |
| Bas (×10$^{9}$/L) | 0.057±0.031 | 0.060±0.036 | 0.605 | 0.855 | **0.051±0.028** | **0.064±0.037** | **0.091** | 0.321 | 0.064±0.033 | 0.055±0.035 | 0.269 | 1.000 |
| Bas (%) | 0.645±0.335 | 0.624±0.314 | 0.686 | 0.807 | 0.612±0.291 | 0.626±0.310 | 0.834 | 0.871 | 0.681±0.379 | 0.621±0.323 | 0.467 | 1.000 |
| Hgb | 131.34±16.05 | 129.54±16.49 | 0.493 | 0.813 | 132.62±18.99 | 130.42±16.54 | 0.560 | 0.733 | 129.92±12.19 | 128.37±16.51 | 0.669 | 1.000 |
| Hct | 0.399±0.045 | 0.387±0.046 | 0.689 | 0.807 | 0.392±0.054 | 0.390±0.046 | 0.850 | 0.852 | 0.387±0.031 | 0.382±0.047 | 0.637 | 1.000 |
| MCV | 85.653±13.243 | 87.300±5.848 | 0.238 | 0.689 | 84.204±17.473 | 87.143±6.230 | 0.205 | 0.551 | 87.269±5.632 | 87.509±5.342 | 0.853 | 0.949 |
| MCH | 29.409±2.632 | 29.235±2.396 | 0.661 | 0.827 | 29.510±2.894 | 29.107±2.538 | 0.485 | 0.710 | 29.296±2.359 | 29.407±2.203 | 0.836 | 0.971 |
| MCHC | 336.818±10.846 | 334.752±9.493 | 0.195 | 1.000 | 337.69±12.54 | 333.85±9.87 | 0.102 | 0.299 | 335.846±8.72 | 335.95±8.92 | 0.962 | 0.962 |
| PLT (×10$^{9}$/L) | 313.182±120.778 | 325.782±11.851 | 0.466 | 0.831 | 300.79±120.72 | 319.84±109.79 | 0.441 | 0.700 | 325.31±121.89 | 333.70±115.04 | 0.763 | 1.000 |
| ALT | 0.334±0.221 | 0.380±0.420 | 0.434 | 0.809 | 0.321±0.160 | 0.3405±0.264 | 0.711 | 0.810 | 0.347±0.274 | 0.434±0.564 | 0.454 | 1.000 |
| AST | 0.371±0.223 | 0.354±0.213 | 0.626 | 0.856 | 0.387±0.242 | 0.333±0.178 | 0.215 | 0.535 | 0.353±0.205 | 0.382±0.252 | 0.598 | 0.987 |
| GGT | 0.818±1.103 | 0.915±2.223 | 0.759 | 0.864 | 1.045±1.390 | 0.758±1.022 | 0.252 | 0.517 | 0.564±0.581 | 1.125±3.196 | 0.378 | 1.000 |
| LDH | 8.040±4.717 | 7.636±5.687 | 0.649 | 0.847 | 8.318±5.466 | 7.501±3.819 | 0.399 | 0.715 | 7.740±3.836 | 7.835±7.696 | 0.953 | 0.962 |
| ALP | 1.606±0.809 | 1.602±0.898 | 0.976 | 0.995 | 1.652±0.829 | 1.537±0.599 | 0.437 | 0.723 | 1.556±0.800 | 1.689±1.188 | 0.602 | 0.987 |
| Total bilirubin | 7.334±3.095 | 7.260±3.705 | 0.895 | 0.948 | 8.069±3.108 | 7.908±4.213 | 0.852 | 0.852 | 6.544±2.934 | 6.395±2.693 | 0.819 | 1.000 |
| Direct bilirubin | 2.582±0.948 | 2.539±1.056 | 0.794 | 0.880 | 2.800±0.975 | 2.701±1.215 | 0.697 | 0.822 | 2.338±0.872 | 2.304±0.721 | 0.830 | 0.979 |
| Urea | 6.104±2.555 | 6.106±2.728 | 0.995 | 0.995 | 6.341±2.195 | 6.588±2.247 | 0.614 | 0.763 | 5.848±2.913 | 5.463±3.169 | 0.595 | 1.000 |
| Serum creatinine | 73.500±20.684 | 71.757±20.280 | 0.592 | 0.855 | 81.448±20.072 | 77.483±18.256 | 0.335 | 0.654 | 64.963±18.027 | 64.123±20.476 | 0.856 | 0.931 |

| | | | | | | | | | | | | |
|---|---|---|---|---|---|---|---|---|---|---|---|---|
| CRP | **26.257±38.730** | **39.659±50.799** | **0.052** | 1.000 | **30.065±42.577** | **49.564±57.749** | **0.065** | 0.310 | 22.165±34.457 | 26.748±36.603 | 0.588 | 1.000 |
| CK | 1.285±0.827 | 1.197±0.982 | 0.561 | 0.879 | 1.402±0.839 | 1.316±1.096 | 0.702 | 0.810 | 1.155±0.811 | 1.08±0.772 | 0.503 | 1.000 |
| CK-MB | 0.426±0.502 | 0.380±0.500 | 0.579 | 0.867 | 0.461±0.494 | 0.368±0.558 | 0.444 | 0.700 | 0.386±0.518 | 0.398±0.396 | 0.916 | 0.963 |
| Uric acid | 313.109±100.258 | 289.565±95.54 | 0.132 | 1.000 | 340.43±101.24 | 307.15±86.23 | 0.099 | 0.299 | 284.78±92.72 | 265.70±102.93 | 0.417 | 1.000 |
| Glucose | 6.452±1.567 | 6.821±2.944 | 0.377 | 0.814 | 6.552±1.611 | 7.100±3.351 | 0.401 | 0.715 | 6.344±1.541 | 6.449±2.267 | 0.829 | 1.000 |

*q-values were obtained using the Benjamini–Hochberg FDR correction for multiple comparisons

**Table S7.** BMI- and age-adjusted *p*-values and Benjamini-Hochberg FDR-adjusted *q*-values for the regression analyses between urinary TCS concentrations (µg/L and µg/gCr) and the observed parameters.

| Parameter | TCS (µg/L) | | | | | | TCS (µg/gCr) | | | | | |
|---|---|---|---|---|---|---|---|---|---|---|---|---|
| | Total N=56 | | Male N=29 | | Female N=27 | | Total N=56 | | Male N=29 | | Female N=27 | |
| | *p*-value | *q*-value | *p*-value | *q*-value | *p*-value | *q*-value | *p*-value | *q*-value | *p*-value | *q*-value | *p*-value | *q*-value |
| WC | 0.431 | 0.764 | 0.444 | 0.866 | 0.788 | 0.975 | 0.199 | 1.000 | 0.382 | 1.000 | 0.881 | 1.000 |
| Hip C | 0.744 | 0.879 | 0.797 | 1.000 | 0.900 | 0.975 | 0.940 | 0.976 | 0.534 | 0.947 | 0.703 | 1.000 |
| WHR | 0.423 | 0.764 | 0.365 | 0.892 | 0.642 | 0.979 | 0.161 | 1.000 | 0.378 | 1.000 | 0.908 | 1.000 |
| WtHR | 0.624 | 0.936 | 0.616 | 1.000 | 0.828 | 0.968 | 0.359 | 1.000 | 0.314 | 1.000 | 0.577 | 1.000 |
| BP systolic | 0.383 | 0.805 | 0.238 | 0.774 | 0.645 | 0.758 | 0.907 | 1.000 | 0.251 | 1.000 | 0.668 | 1.000 |
| BP diastolic | 0.909 | 0.951 | 0.361 | 0.949 | 0.272 | 0.758 | 0.236 | 1.000 | 0.271 | 1.000 | 0.198 | 1.000 |
| Pulse | 0.223 | 1.000 | 0.923 | 1.000 | 0.241 | 0.730 | **0.046** | 1.000 | 0.924 | 0.983 | 0.141 | 0.983 |
| RBC ($\times 10^{12}$/L) | 0.301 | 0.894 | 0.479 | 0.890 | 0.393 | 0.458 | 0.815 | 0.997 | 0.945 | 0.950 | 0.933 | 0.983 |
| WBC ($\times 10^{9}$/L) | **0.078** | 1.000 | 0.205 | 0.945 | **0.047** | 0.458 | 0.479 | 1.000 | 0.912 | 1.000 | 0.210 | 1.000 |
| Neu ($\times 10^{9}$/L) | 0.136 | 0.910 | 0.237 | 0.840 | 0.172 | 0.745 | 0.410 | 1.000 | 0.795 | 0.970 | 0.266 | 1.000 |
| Neu (%) | 0.663 | 0.958 | 0.389 | 0.843 | 0.471 | 0.468 | 0.340 | 1.000 | 0.321 | 1.000 | 0.840 | 1.000 |
| Lym ($\times 10^{9}$/L) | 0.218 | 1.000 | 0.420 | 0.862 | **0.036** | 0.468 | 0.818 | 0.997 | 0.740 | 0.974 | 0.448 | 0.988 |
| Lym (%) | 0.978 | 0.978 | 0.743 | 1.000 | 0.352 | 0.723 | 0.632 | 1.000 | 0.458 | 1.000 | 0.963 | 0.988 |
| Mon ($\times 10^{9}$/L) | 0.392 | 0.803 | 0.862 | 0.961 | 0.168 | 0.819 | 0.415 | 1.000 | 0.419 | 1.000 | 0.790 | 1.000 |
| Mon (%) | 0.321 | 0.868 | 0.155 | 0.886 | 0.826 | 0.860 | **0.099** | 1.000 | 0.243 | 1.000 | 0.338 | 1.000 |
| Eos ($\times 10^{9}$/L) | 0.701 | 0.944 | 0.546 | 0.968 | 0.507 | 0.860 | 0.934 | 0.991 | 0.950 | 0.950 | 0.576 | 0.999 |
| Eos (%) | 0.345 | 0.791 | 0.379 | 0.843 | 0.913 | 0.696 | 0.614 | 1.000 | 0.688 | 0.958 | 0.999 | 0.999 |
| Bas ($\times 10^{9}$/L) | 0.412 | 0.786 | 0.649 | 1.000 | 0.125 | 0.696 | **0.097** | 1.000 | 0.275 | 1.000 | **0.018** | 0.702 |
| Bas (%) | 0.927 | 0.951 | 0.859 | 0.961 | 0.640 | 0.961 | 0.371 | 1.000 | 0.590 | 0.920 | 0.182 | 1.000 |
| Hgb | **0.043** | 0.839 | **0.055** | 1.000 | 0.862 | 0.961 | 0.651 | 0.940 | 0.458 | 1.000 | 0.706 | 1.000 |
| Hct | **0.034** | 0.839 | **0.057** | 0.754 | 0.961 | 0.961 | 0.635 | 1.000 | 0.484 | 0.993 | 0.817 | 1.000 |
| MCV | 0.235 | 0.936 | 0.237 | 0.774 | 0.296 | 0.770 | 0.367 | 1.000 | 0.517 | 0.973 | 0.839 | 1.000 |
| MCH | 0.128 | 1.000 | **0.058** | 0.754 | 0.269 | 0.792 | 0.346 | 1.000 | 0.266 | 1.000 | 0.679 | 1.000 |
| MCHC | 0.896 | 0.958 | 0.805 | 0.995 | 0.325 | 0.792 | 0.999 | 0.999 | 0.814 | 0.934 | 0.431 | 1.000 |
| PLT ($\times 10^{9}$/L) | 0.240 | 0.936 | 0.096 | 0.936 | 0.676 | 0.976 | 0.459 | 1.000 | 0.130 | 1.000 | 0.746 | 0.993 |
| ALT | 0.888 | 0.971 | 0.850 | 0.985 | 0.738 | 0.694 | 0.647 | 0.940 | 0.387 | 1.000 | 0.917 | 0.993 |
| AST | 0.479 | 0.812 | 0.979 | 0.983 | 0.356 | 0.694 | 0.758 | 0.985 | 0.641 | 0.962 | 0.912 | 1.000 |
| GGT | 0.140 | 0.910 | 0.144 | 1.000 | 0.724 | 0.507 | 0.682 | 0.925 | 0.933 | 0.970 | 0.808 | 1.000 |
| LDH | 0.339 | 0.791 | 0.366 | 0.869 | **0.065** | 0.507 | 0.879 | 1.000 | 0.583 | 0.920 | 0.484 | 1.000 |
| ALP | 0.826 | 0.947 | 0.974 | 1.000 | 0.343 | 0.741 | 0.209 | 1.000 | 0.471 | 0.993 | 0.800 | 1.000 |
| Total bilirubin | 0.334 | 0.826 | 0.752 | 1.000 | 0.190 | 0.429 | 0.618 | 1.000 | 0.677 | 0.958 | 0.460 | 1.000 |
| Direct bilirubin | 0.535 | 0.869 | 0.983 | 0.983 | **0.022** | 0.429 | 0.644 | 0.971 | 0.567 | 0.947 | 0.120 | 1.000 |
| Urea | 0.729 | 0.896 | 0.816 | 0.995 | 0.337 | 0.773 | 0.895 | 1.000 | 0.524 | 0.947 | 0.294 | 1.000 |
| Serum creatinine | 0.581 | 0.906 | 0.218 | 0.924 | 0.270 | 0.462 | 0.424 | 1.000 | 0.334 | 1.000 | 0.398 | 1.000 |
| CRP | 0.702 | 0.922 | 0.278 | 0.834 | **0.071** | 0.462 | 0.623 | 1.000 | 0.371 | 1.000 | 0.254 | 1.000 |
| CK | 0.735 | 0.879 | 0.677 | 0.979 | 0.946 | 0.971 | 0.688 | 0.925 | 0.796 | 0.949 | 0.792 | 0.689 |
| CK-MB | 0.266 | 0.943 | 0.159 | 0.886 | 0.753 | 0.312 | 0.336 | 1.000 | 0.803 | 0.934 | **0.053** | 0.689 |
| Uric acid | 0.298 | 0.903 | 0.678 | 0.979 | **0.008** | 0.312 | 0.495 | 1.000 | 0.431 | 1.000 | **0.045** | 0.878 |
| Glucose | 0.709 | 0.917 | 0.584 | 0.990 | 0.851 | 0.976 | 0.951 | 0.976 | 0.749 | 0.974 | 0.559 | 1.000 |

**Table S8.** Comparison of analyzed parameters between patients with urinary RCO levels above and below the LOQ in the total cohort and stratified by sex.

| | **Total** | | | | **Male** | | | | **Female** | | | |
|---|---|---|---|---|---|---|---|---|---|---|---|---|
| Parameter | RCO ≥LOQ N=22 | RCO <LOQ N=168 | *p*-value | *q*-value* | RCO ≥LOQ N=15 | RCO <LOQ N=90 | *p*-value | *q*-value* | RCO ≥LOQ N=7 | RCO <LOQ N=78 | *p*-value | *q*-value* |
| Age | 66.636±7.932 | 65.542±8.613 | 0.572 | 0.838 | 65.467±7.357 | 67.322±8.554 | 0.430 | 0.705 | 69.143±9.118 | 63.487±8.266 | **0.089** | 1.000 |
| BMI | 23.706±4.301 | 25.035±4.870 | 0.225 | 1.000 | 24.373±4.763 | 25.119±4.301 | 0.542 | 0.794 | 22.279±2.886 | 24.938±5.482 | 0.210 | 0.957 |
| WC | 91.364±14.325 | 91.275±14.549 | 0.979 | 0.980 | 95.600±14.653 | 94.847±13.358 | 0.845 | 0.990 | 82.286±8.731 | 87.227±14.649 | 0.384 | 0.984 |
| Hip C | 97.182±8.754 | 98.444±11.110 | 0.610 | 0.807 | 98.267±9.153 | 97.212±9.655 | 0.965 | 0.965 | 94.857±7.967 | 99.840±12.477 | 0.304 | 1.000 |
| WHR | 0.937±0.093 | 0.927±0.102 | 0.641 | 0.821 | 0.970±0.091 | 0.974±0.081 | 0.875 | 0.991 | 0.867±0.047 | 0.873±0.096 | 0.864 | 0.973 |
| WtHR | 0.541±0.082 | 0.521±0.131 | 0.501 | 0.801 | 0.554±0.091 | 0.519±0.136 | 0.339 | 0.777 | 0.5112±0.051 | 0.524±0.124 | 0.792 | 1.000 |
| BP systolic | 121.134±17.656 | 125.611±18.325 | 0.282 | 0.964 | 119.33±13.74 | 127.54±18.67 | 0.107 | 1.000 | 125.00±25.00 | 123.37±17.78 | 0.824 | 0.998 |
| BP diastolic | 70.025±11.981 | 73.025±10.278 | 0.250 | 1.000 | 70.00±10.18 | 72.80±10.49 | 0.341 | 0.751 | 70.857±16.13 | 73.28±10.09 | 0.567 | 1.000 |
| Pulse | 84.228±10.555 | 80.605±15.166 | 0.280 | 0.964 | **85.60±11.59** | **79.07±14.31** | **0.097** | 1.000 | 81.29±7.81 | 82.387±16.02 | 0.858 | 0.984 |
| RBC ($\times 10^{12}$/L) | 4.520±0.329 | 4.463±0.605 | 0.665 | 0.826 | 4.609±0.333 | 4.518±0.698 | 0.618 | 0.874 | 4.330±0.238 | 4.399±0.467 | 0.700 | 1.000 |
| WBC ($\times 10^{9}$/L) | 10.086±2.589 | 9.980±6.984 | 0.944 | 0.992 | 10.280±2.624 | 9.560±3.168 | 0.407 | 0.711 | 9.671±2.665 | 10.479±9.743 | 0.828 | 0.998 |
| Neu ($\times 10^{9}$/L) | 7.265±2.198 | 6.883±3.363 | 0.606 | 0.807 | 7.282±2.222 | 7.003±2.885 | 0.723 | 0.972 | 7.229±2.319 | 6.741±3.870 | 0.745 | 1.000 |
| Neu (%) | 71.409±7.049 | 70.668±9.081 | 0.713 | 0.856 | 70.067±6.954 | 71.770±8.596 | 0.469 | 0.740 | 74.286±6.849 | 69.360±9.518 | 0.186 | 0.953 |
| Lym ($\times 10^{9}$/L) | 1.720±0.581 | 1.742±0.728 | 0.891 | 0.961 | 1.768±0.531 | 1.591±0.640 | 0.314 | 0.848 | 1.617±0.711 | 1.922±0.787 | 0.327 | 0.961 |
| Lym (%) | 17.410±5.369 | 19.362±8.083 | 0.273 | 1.000 | 17.600±4.997 | 17.499±7.458 | 0.960 | 0.989 | 17.00±6.51 | 21.573±8.284 | 0.160 | 0.937 |
| Mon ($\times 10^{9}$/L) | **0.849±0.276** | **0.724±0.303** | **0.073** | 1.000 | **0.925±0.249** | **0.778±0.271** | **0.051** | 1.000 | 0.679±0.273 | 0.661±0.327 | 0.887 | 0.957 |
| Mon (%) | 8.455±2.385 | 7.746±2.354 | 0.187 | 1.000 | 9.133±2.134 | 8.295±2.308 | 0.192 | 0.875 | 7.00±2.38 | 7.09±2.55 | 0.917 | 0.948 |
| Eos ($\times 10^{9}$/L) | 0.193±0.155 | 0.147±0.236 | 0.373 | 0.861 | 0.241±0.161 | 0.158±0.304 | 0.370 | 0.697 | 0.091±0.079 | 0.122±0.108 | 0.476 | 1.000 |
| Eos (%) | 1.873±1.395 | 1.544±2.027 | 0.462 | 0.818 | 2.307±1.434 | 1.672±2.532 | 0.348 | 0.720 | 0.943±0.730 | 1.392±1.178 | 0.326 | 1.000 |
| Bas ($\times 10^{9}$/L) | 0.700±0.046 | 0.058±0.033 | 0.240 | 1.000 | 0.073±0.048 | 0.059±0.032 | 0.154 | 0.902 | 0.064±0.044 | 0.056±0.034 | 0.597 | 0.943 |
| Bas (%) | 0.673±0.408 | 0.625±0.307 | 0.509 | 0.773 | 0.693±0.425 | 0.611±0.279 | 0.331 | 0.818 | 0.629±0.399 | 0.641±0.338 | 0.925 | 0.948 |
| Hgb | 132.909±14.369 | 129.693±16.592 | 0.387 | 0.834 | 136.53±13.92 | 130.11±17.57 | 0.181 | 0.875 | 125.14±12.93 | 129.19±15.49 | 0.504 | 1.000 |
| Hct | 0.397±0.038 | 0.387±0.047 | 0.337 | 0.962 | 0.408±0.04 | 0.388±0.049 | 0.153 | 0.902 | 0.373±0.031 | 0.385±0.044 | 0.506 | 1.000 |
| MCV | 87.727±4.641 | 86.698±9.084 | 0.602 | 0.828 | 88.400±5.193 | 85.987±11.212 | 0.416 | 0.705 | 86.286±2.984 | 87.539±5.572 | 0.560 | 1.000 |
| MCH | 29.377±1.973 | 29.274±2.524 | 0.854 | 0.946 | 29.627±2.105 | 29.150±2.715 | 0.519 | 0.788 | 28.843±1.673 | 29.421±2.286 | 0.517 | 1.000 |
| MCHC | 334.909±7.874 | 335.416±10.183 | 0.823 | 0.937 | 334.80±7.30 | 334.93±11.25 | 0.965 | 0.965 | 335.14±9.62 | 335.99±8.79 | 0.810 | 1.000 |
| PLT ($\times 10^{9}$/L) | 329.773±132.363 | 320.813±112.186 | 0.731 | 0.856 | 312.80±134.35 | 314.88±109.49 | 0.948 | 1.000 | 336.14±130.15 | 327.84±115.63 | 0.409 | 0.986 |
| ALT | 0.489±0.677 | 0.349±0.308 | 0.104 | 1.000 | 0.393±0.274 | 0.325±0.232 | 0.308 | 0.858 | 0.687±1.166 | 0.379±0.377 | 0.110 | 1.000 |
| AST | 0.391±0.287 | 0.354±0.204 | 0.451 | 0.824 | 0.342±0.183 | 0.349±0.202 | 0.894 | 0.991 | 0.497±0.437 | 0.360±0.209 | 0.144 | 0.937 |
| GGT | 0.626±0.578 | 7.019±75.497 | 0.508 | 0.773 | 0.585±0.226 | 0.883±1.224 | 0.351 | 0.720 | 0.714±1.017 | 0.967±2.767 | 0.812 | 1.000 |
| LDH | 6.865±4.126 | 7.889±5.554 | 0.407 | 0.834 | 8.081±4.356 | 7.667±4.347 | 0.735 | 0.956 | 4.260±1.922 | 8.157±6.793 | 0.136 | 0.984 |
| ALP | 1.599±0.714 | 2.604±0.889 | 0.980 | 0.980 | 1.519±0.468 | 1.579±0.702 | 0.758 | 0.942 | 1.785±1.144 | 1.633±1.071 | 0.739 | 1.000 |
| Total bilirubin | 6.341±2.767 | 7.406±3.604 | 0.184 | 1.000 | 7.113±3.008 | 8.092±4.055 | 0.374 | 0.697 | **4.686±1.001** | **6.603±2.812** | **0.078** | 1.000 |
| Direct bilirubin | 2.317±0.639 | 2.584±1.062 | 0.245 | 1.000 | 2.440±0.725 | 2.778±1.203 | 0.295 | 0.902 | 2.043±0.282 | 2.342±0.797 | 0.329 | 0.899 |
| Urea | 5.632±1.954 | 6.168±2.751 | 0.378 | 0.835 | 5.933±1.864 | 6.618±2.274 | 0.272 | 0.977 | 4.986±2.130 | 5.642±3.154 | 0.592 | 0.979 |
| Serum creatinine | 67.364±14.311 | 72.921±20.975 | 0.230 | 1.000 | 72.067±12.050 | 79.663±19.497 | 0.147 | 1.000 | 57.286±14.291 | 65.039±19.977 | 0.320 | 1.000 |

| | | | | | | | | | | | | |
|---|---|---|---|---|---|---|---|---|---|---|---|---|
| CRP | 28.629±30.337 | 36.495±49.554 | 0.479 | 0.818 | **30.221±26.103** | **46.216±57.418** | **0.093** | 1.000 | 25.443±39.67 | 25.240±35.864 | 0.989 | 0.989 |
| CK | 1.407±1.184 | 1.198±0.898 | 0.328 | 0.987 | 1.635±1.364 | 1.290±0.958 | 0.231 | 0.947 | 0.920±0.402 | 1.085±0.810 | 0.598 | 0.943 |
| CK-MB | 0.316±0.274 | 0.405±0.524 | 0.434 | 0.841 | 0.352±0.329 | 0.401±0.571 | 0.746 | 0.942 | 0.390±0.035 | 0.411±0.460 | 0.328 | 0.899 |
| Uric acid | 279.0.46±85.798 | 298.816±98.709 | 0.372 | 0.900 | 310.80±60.452 | 317.007±95.67 | 0.809 | 0.976 | **211.00±96.51** | **277.51±98.56** | **0.091** | 1.000 |
| Glucose | 6.223±1.680 | 6.776±2.710 | 0.352 | 0.953 | 6.189±1.196 | 7.076±3.164 | 0.286 | 0.930 | 6.300±2.555 | 6.426±2.021 | 0.878 | 0.957 |

*q-values were obtained using the Benjamini–Hochberg FDR correction for multiple comparisons

**Table S9.** BMI- and age-adjusted *p*-values and Benjamini-Hochberg FDR-adjusted *q*-values for the regression analyses between urinary RCO concentrations (µg/L and µg/gCr) and the observed parameters.

| Parameter | RCO (µg/L) | | | | | | RCO (µg/gCr) | | | | | |
|---|---|---|---|---|---|---|---|---|---|---|---|---|
| | Total N=22 | | Male N=15 | | Female N=7 | | Total N=22 | | Male N=15 | | Female N=7 | |
| | *p*-value | *q*-value | *p*-value | *q*-value | *p*-value | *q*-value | *p*-value | *q*-value | *p*-value | *q*-value | *p*-value | *q*-value |
| WC | 0.111 | 1.000 | **0.085** | 0.878 | **0.014** | 0.546 | **0.053** | 1.000 | **0.075** | 1.000 | 0.445 | 0.765 |
| Hip C | 0.992 | 0.992 | 0.693 | 1.000 | 0.832 | 0.954 | 0.925 | 0.975 | 0.719 | 1.000 | 0.454 | 0.738 |
| WHR | 0.112 | 1.000 | 0.126 | 0.819 | 0.413 | 1.000 | **0.064** | 0.871 | 0.108 | 0.809 | 0.266 | 0.834 |
| WtHR | 0.185 | 1.000 | **0.092** | 0.718 | 0.165 | 0.839 | 0.226 | 0.893 | **0.083** | 0.809 | 0.618 | 0.689 |
| BP systolic | 0.968 | 1.000 | 0.673 | 1.000 | 0.132 | 0.858 | 0.560 | 0.891 | 0.903 | 1.000 | 0.598 | 0.722 |
| BP diastolic | 0.574 | 1.000 | 0.262 | 1.000 | 0.457 | 0.969 | 0.898 | 1.000 | 0.318 | 1.000 | 0.868 | 0.868 |
| Pulse | 0.367 | 1.000 | 0.456 | 1.000 | 0.119 | 0.858 | 0.663 | 0.892 | 0.538 | 1.000 | 0.577 | 0.726 |
| RBC ($\times10^{12}$/L) | 0.985 | 0.992 | 0.837 | 1.000 | 0.922 | 0.992 | 0.350 | 1.000 | 0.921 | 1.000 | 0.736 | 0.776 |
| WBC ($\times10^{9}$/L) | 0.438 | 1.000 | 0.307 | 1.000 | 0.472 | 0.922 | 0.708 | 0.920 | 0.235 | 1.000 | 0.618 | 0.689 |
| Neu ($\times10^{9}$/L) | 0.596 | 1.000 | 0.360 | 1.000 | 0.321 | 1.000 | 0.988 | 0.988 | 0.357 | 1.000 | 0.432 | 0.804 |
| Neu (%) | 0.723 | 1.000 | 0.875 | 1.000 | 0.088 | 0.858 | 0.404 | 0.934 | 0.804 | 0.579 | **0.061** | 1.000 |
| Lym ($\times10^{9}$/L) | 0.187 | 1.000 | 0.406 | 1.000 | 0.441 | 0.990 | **0.067** | 0.839 | 0.178 | 0.579 | 0.369 | 0.996 |
| Lym (%) | 0.546 | 1.000 | 0.853 | 1.000 | **0.065** | 0.936 | 0.129 | 0.969 | 0.814 | 1.000 | **0.095** | 0.926 |
| Mon ($\times10^{9}$/L) | 0.984 | 1.000 | 0.736 | 1.000 | 0.192 | 0.832 | 0.512 | 0.908 | 0.440 | 1.000 | 0.120 | 0.852 |
| Mon (%) | 0.515 | 1.000 | 0.606 | 1.000 | 0.428 | 1.000 | 0.229 | 0.823 | 0.883 | 0.909 | **0.095** | 0.926 |
| Eos ($\times10^{9}$/L) | 0.603 | 1.000 | 0.434 | 1.000 | 0.593 | 0.939 | 0.809 | 0.998 | 0.373 | 0.909 | 0.253 | 0.865 |
| Eos (%) | 0.915 | 1.000 | 0.681 | 1.000 | 0.813 | 0.954 | 0.914 | 0.999 | 0.666 | 0.767 | 0.131 | 0.852 |
| Bas ($\times10^{9}$/L) | 0.227 | 1.000 | 0.412 | 1.000 | 0.602 | 0.905 | 0.157 | 0.875 | 0.354 | 0.767 | 0.226 | 0.979 |
| Bas (%) | 0.384 | 1.000 | 0.702 | 1.000 | 0.405 | 1.000 | 0.210 | 0.979 | 0.668 | 1.000 | **0.071** | 1.000 |
| Hgb | 0.977 | 1.000 | 0.763 | 1.000 | 0.943 | 0.968 | 0.369 | 1.000 | 0.840 | 1.000 | 0.451 | 0.738 |
| Hct | 0.931 | 1.000 | 0.674 | 1.000 | 0.902 | 0.999 | 0.407 | 0.921 | 0.776 | 0.931 | 0.611 | 0.709 |
| MCV | 0.830 | 1.000 | 0.651 | 1.000 | 0.624 | 0.891 | 0.819 | 0.998 | 0.525 | 0.931 | 0.433 | 0.789 |
| MCH | 0.910 | 1.000 | 0.773 | 1.000 | 0.810 | 0.961 | 0.649 | 0.892 | 0.686 | 1.000 | 0.278 | 0.834 |
| MCHC | 0.890 | 1.000 | 0.812 | 1.000 | 0.941 | 0.968 | 0.461 | 0.856 | 0.857 | 1.000 | 0.172 | 0.839 |
| PLT ($\times10^{9}$/L) | 0.977 | 1.000 | 0.826 | 1.000 | 0.750 | 0.944 | 0.958 | 0.983 | 0.935 | 0.120 | 0.388 | 0.911 |
| ALT | 0.239 | 1.000 | **0.090** | 0.718 | 0.529 | 0.938 | 0.240 | 0.780 | 0.080 | 0.019 | 0.490 | 0.746 |
| AST | **0.060** | 1.000 | **0.010** | 0.390 | 0.648 | 0.871 | **0.086** | 0.839 | **0.013** | 0.019 | 0.497 | 0.722 |
| GGT | 0.508 | 1.000 | 0.429 | 1.000 | 0.473 | 0.922 | 0.644 | 0.904 | 0.495 | 0.689 | 0.421 | 0.842 |
| LDH | 0.626 | 1.000 | 0.853 | 1.000 | 0.603 | 0.901 | 0.428 | 0.860 | 0.827 | 0.647 | 0.252 | 0.897 |
| ALP | 0.271 | 1.000 | 0.620 | 1.000 | 0.172 | 0.832 | 0.393 | 0.985 | 0.498 | 0.647 | 0.400 | 0.864 |
| Total bilirubin | 0.816 | 1.000 | 0.906 | 0.983 | 0.384 | 1.000 | 0.624 | 0.930 | 0.730 | 0.896 | 0.397 | 0.867 |
| Direct bilirubin | 0.972 | 1.000 | 0.956 | 0.957 | 0.399 | 1.000 | 0.441 | 0.856 | 0.735 | 0.229 | 0.500 | 0.722 |
| Urea | 0.335 | 1.000 | **0.078** | 1.000 | 0.520 | 0.938 | 0.873 | 1.000 | 0.194 | 0.229 | 0.383 | 0.946 |
| Serum creatinine | 0.973 | 1.000 | 0.953 | 0.981 | **0.072** | 0.858 | 0.425 | 0.879 | 0.774 | 0.349 | 0.668 | 0.724 |
| CRP | 0.362 | 1.000 | 0.313 | 1.000 | 0.374 | 1.000 | 0.552 | 0.910 | 0.313 | 0.349 | 0.540 | 0.748 |
| CK | 0.616 | 1.000 | 0.561 | 1.000 | 0.672 | 0.874 | 0.922 | 0.975 | 0.696 | 0.565 | 0.763 | 0.783 |
| CK-MB | 0.969 | 1.000 | 0.327 | 1.000 | 0.640 | 0.871 | 0.571 | 0.891 | 0.536 | 0.565 | 0.569 | 0.726 |
| Uric acid | 0.492 | 1.000 | 0.957 | 0.957 | 0.571 | 0.964 | 0.149 | 0.875 | 0.596 | 0.612 | 0.162 | 0.839 |
| Glucose | 0.550 | 1.000 | 0.907 | 0.983 | 0.982 | 0.982 | 0.232 | 0.780 | 0.685 | 0.685 | 0.556 | 0.740 |

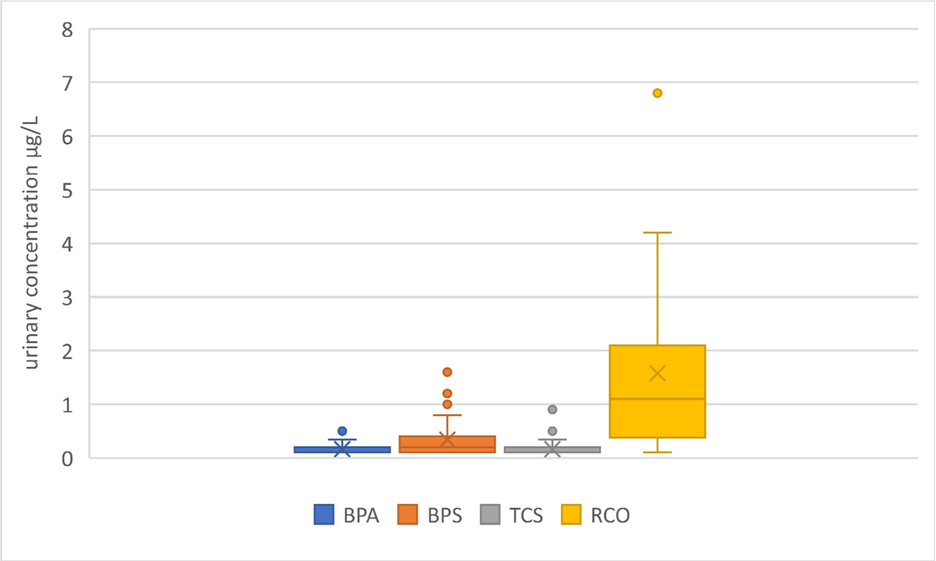


(a)

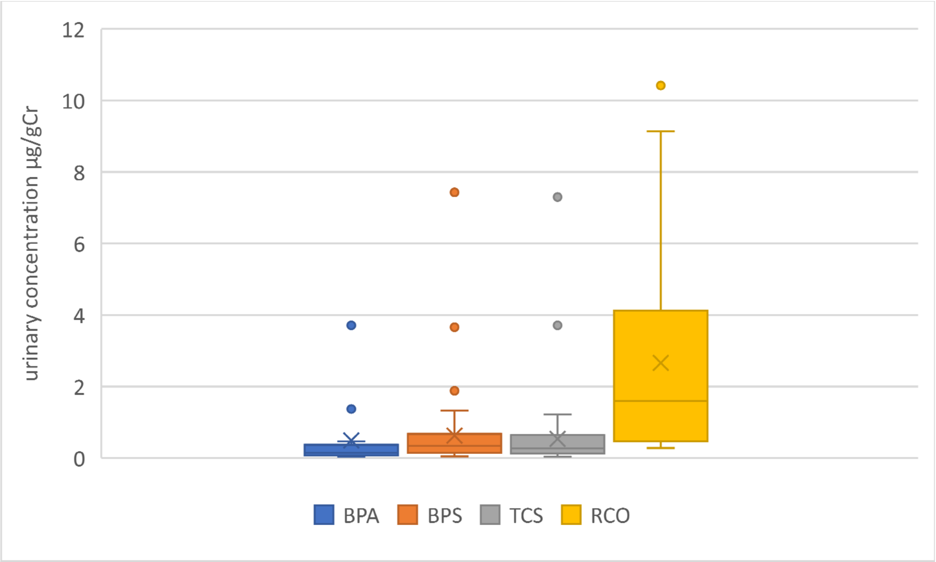


(b)

**Figure S1.** Box plot of urinary concentrations of the selected EDCs in (a) µg/L and (b) µg/gCr.
